# Influence of Varying Dietary ω6 to ω3 Fatty Acid Ratios on the Hepatic Transcriptome, and Association with Phenotypic Traits (Growth, Somatic Indices, and Tissue Lipid Composition), in Atlantic Salmon (*Salmo salar*)

**DOI:** 10.3390/biology10070578

**Published:** 2021-06-24

**Authors:** Tomer Katan, Xi Xue, Albert Caballero-Solares, Richard G. Taylor, Christopher C. Parrish, Matthew L. Rise

**Affiliations:** 1 Department of Ocean Sciences, Memorial University of Newfoundland, St. John’s, NL A1C 5S7, Canada; xi.xue@mun.ca (X.X.); cparrish@mun.ca (C.C.P.); mrise@mun.ca (M.L.R.); 2 Cargill Animal Nutrition, 10383 165th Avenue NW, Elk River, MN 55330, USA; richard_taylor@cargill.com

**Keywords:** hepatic transcript expression, lipid metabolism, salmon, microarray, omega-6/omega-3 ratio, nutrigenomics, fatty acids, liver, muscle

## Abstract

**Simple Summary:**

Plant oils are routinely used in fish feeds as a fish oil replacement. However, these terrestrial alternatives typically contain high levels of ω6 fatty acids (FA) and, thus, high ω6 to ω3 (ω6:ω3) FA ratios, which influence farmed fish and their consumers. The ω6:ω3 ratio is known to affect many biological processes (e.g., inflammation, FA metabolism) and human diseases; however, its impacts on fish physiology and the underlying molecular mechanisms are less well understood. In this study, we used 44 K microarrays to examine which genes and molecular pathways are altered by variation in dietary ω6:ω3 in Atlantic salmon. Our microarray study showed that several genes related to immune response, lipid metabolism, cell proliferation, and translation were differentially expressed between the two extreme ω6:ω3 dietary treatments. We also revealed that the PPARα activation-related transcript *helz2* is a potential novel molecular biomarker of tissue variation in ω6:ω3. Further, correlation analyses illustrated the relationships between liver transcript expression and tissue (liver, muscle) lipid composition, and other phenotypic traits in salmon fed low levels of fish oil. This nutrigenomic study enhanced the current understanding of Atlantic salmon gene expression response to varying dietary ω6:ω3.

**Abstract:**

The importance of dietary omega-6 to omega-3 (ω6:ω3) fatty acid (FA) ratios for human health has been extensively examined. However, its impact on fish physiology, and the underlying molecular mechanisms, are less well understood. This study investigated the influence of plant-based diets (12-week exposure) with varying ω6:ω3 (0.4–2.7) on the hepatic transcriptome of Atlantic salmon. Using 44 K microarray analysis, genes involved in immune and inflammatory response (*lect2a*, *itgb5*, *helz2a*, *p43*), lipid metabolism (*helz2a*), cell proliferation (*htra1b*), control of muscle and neuronal development (*mef2d*) and translation (*eif2a*, *eif4b1*, *p43*) were identified; these were differentially expressed between the two extreme ω6:ω3 dietary treatments (high ω6 vs. high ω3) at week 12. Eight out of 10 microarray-identified transcripts showed an agreement in the direction of expression fold-change between the microarray and qPCR studies. The PPARα activation-related transcript *helz2a* was confirmed by qPCR to be down-regulated by high ω6 diet compared with high ω3 diet. The transcript expression of two *helz2* paralogues was positively correlated with ω3, and negatively with ω6 FA in both liver and muscle, thus indicating their potential as biomarkers of tissue ω6:ω3 variation. *Mef2d* expression in liver was suppressed in the high ω6 compared to the balanced diet (ω6:ω3 of 2.7 and 0.9, respectively) fed fish, and showed negative correlations with ω6:ω3 in both tissues. The hepatic expression of two *lect2* paralogues was negatively correlated with viscerosomatic index, while *htra1b* correlated negatively with salmon weight gain and condition factor. Finally, *p43* and *eif2a* were positively correlated with liver Σω3, while these transcripts and *eif4b2* showed negative correlations with 18:2ω6 in the liver. This suggested that some aspects of protein synthesis were influenced by dietary ω6:ω3. In summary, this nutrigenomic study identified hepatic transcripts responsive to dietary variation in ω6:ω3, and relationships of transcript expression with tissue (liver, muscle) lipid composition and other phenotypic traits.

## 1. Introduction

Plant-based oils are commonly used in aquafeeds to replace fish oil (FO), due to decreasing global availability, rising market price, and concerns regarding the ecological sustainability of the finite fishery resources upon which FO production depends [1,2]. Indeed, plant oils (PO) were shown to be more economical and environmentally sustainable [3], and their inclusion as alternatives to FO in many experimental diets did not affect the growth and survival of farmed Atlantic salmon (*Salmo salar*) [4,5,6]. However, terrestrial oils are devoid of long-chain polyunsaturated fatty acids (LC-PUFA), such as eicosapentaenoic acid (EPA, 20:5ω3), docosahexaenoic acid (DHA, 22:6ω3), and arachidonic acid (ARA, 20:4ω6), which are abundant in FO. These LC-PUFA have important functions in vertebrate health, reproduction, neural development, and growth, among other biological processes [5,7]. This has resulted in decreased fillet EPA and DHA levels in farmed fish that were fed with PO as a partial or full replacement for FO, compromising their nutritional quality for human consumers [8,9,10]. Further, previous studies reported impacts on fish health and physiology with the dietary replacement of FO by PO (e.g., liver steatosis, altered complement pathway and phagocytic activity, and modulated expression of genes involved in immune response) [11,12,13,14,15]. Another concern is that most terrestrial oils used in aquafeeds, and the farmed seafood consuming them, may not provide adequate ratios of ω6 to ω3 (ω6:ω3) fatty acids (FA) due to high ω6 FA content [10,16,17,18]. Previous human nutrition studies reported that high dietary ω6:ω3 promotes the pathogenesis of many diseases, including cardiovascular, inflammatory, autoimmune, and cognitive diseases, as well as obesity and cancer [19,20,21,22]. An optimal ratio of ω6:ω3 is important for maintaining the homeostasis of many biological processes such as cell apoptosis, inflammation, fatty acid and cholesterol metabolism, and others [23,24,25]. However, the underlying molecular mechanisms are still poorly understood in fish, and it is not known which genes are involved in variation in dietary and tissue ω6:ω3 in salmon fed high levels of terrestrial-based oils.

A feeding trial was performed to examine the impact of five plant-based diets with varying ω6:ω3 on salmon growth, tissue (i.e., muscle, liver) lipid composition, liver LC-PUFA synthesis, and transcript expression (targeted qPCR) of lipid metabolism and eicosanoid synthesis-related genes [26]. The objective of our current study was to utilize a 44 K salmonid oligonucleotide microarray [27,28,29] for the examination of the impact of the two extreme ω6:ω3 diets (i.e., high ω6 and high ω3) on the hepatic transcriptome at week 12. We hypothesized that salmon fed the two diets with the most extreme lipid compositions (i.e., High ω3 and High ω6) would show the most extensive transcriptomic differences. The current study used the same fish as in Katan et al. [26]. The aim was to identify novel biomarker genes and molecular pathways that are altered by variation in ω6:ω3. To aid in the elucidation of the relationships between liver transcripts and phenotypic traits (i.e., growth parameters, somatic indices, tissue FA and lipid class composition), correlation analyses were also performed.

## 2. Materials and Methods

### 2.1. Fish and Experimental Diets

Five experimental diets with varying ratios of ω6:ω3 were formulated and manufactured by Cargill Innovation Center (Dirdal, Norway). The diets had ω6:ω3 of 1:3 (high ω3), 1:2 (medium ω3), 1:1 (balanced), 2:1 (medium ω6) and 3:1 (high ω6). Dietary formulations and their lipid composition were published previously [26]. However, as they pertain to the current study, the formulation and lipid profiles of the relevant diets (i.e., high ω3, balanced, and high ω6) are also included as supplementary material herein (Tables S1 and S2). All diets contained the same sources and equal levels of marine and plant proteins, but had different mixes of plant-based oils (i.e., linseed (flax), soy, and palm). All diets were formulated to be isonitrogenous and isoenergetic (Table S1), and to meet the nutritional requirements of salmonids [30].

Atlantic salmon pre-smolts were transported from Northern Harvest Sea Farms (Stephenville, NL, Canada) in October 2015, and held in the Dr. Joe Brown Aquatic Research Building (Ocean Sciences Centre, Memorial University of Newfoundland, St. John’s, NL, Canada) in 3800-L tanks. After their arrival, fish were graded in order to select the most uniform population, and this was followed by PIT (Passive Integrated Transponder; Easy AV, Avid Identification Systems, Norco, CA, USA)-tagging for individual identification. Then, post-smolts (203 ± 24 g mean initial weight ± SE) were randomly distributed into twenty 620-L tanks (40 fish tank^−1^), and subjected to a 2.5-week acclimation period. After the completion of the acclimation period, fish were switched from the commercial diet (Nutra Transfer NP, 3 mm, Skretting Canada, St. Andrews, NB, Canada), and fed with the experimental diets (4 tanks diet^−1^) for 12 weeks. The photoperiod was maintained at 24 h light. Fish were fed overnight using automatic feeders, and apparent feed intake was recorded throughout the trial. Mortalities were also recorded during the trial. For additional details regarding the rearing conditions and recordings, refer to Katan et al. [26].

### 2.2. Sample Collection

Growth performance parameters (e.g., fork-length, weight, organ indices) were measured at the beginning and the end of the 12-week feeding trial [26]. At the end of the trial, salmon were starved for 24 h, and then 5 fish per tank were euthanized with an overdose of MS-222 (400 mg L^−1^; Syndel Laboratories, Vancouver, BC, Canada) and dissected for tissue collection. For gene expression analyses, liver samples (50–100 mg) were collected in 1.5 mL nuclease-free tubes, flash-frozen in liquid nitrogen, and stored at −80 °C until RNA extractions were performed. Liver and muscle samples, for lipid analyses, were collected, processed, and stored as described in Katan et al. [26]. Only liver samples from fish that showed weight gains within one standard deviation below and above the mean value of each tank were utilized for this study, in order to reduce biological variability in the gene expression data among fish. Tank means rather than dietary treatment means, were chosen for sample selection, so that variability between tanks could be included in the statistical analysis.

### 2.3. RNA Extraction, DNase Treatment, Column Purification and cDNA Synthesis

The TissueLyser Ⅱ system (at 25 Hz for 2.5 min) with 5 mm stainless steel beads, QIAGEN, Mississauga, ON, Canada) was used to homogenize liver samples in TRIzol reagent (Invitrogen, Carlsbad, CA, USA). Samples were subjected to RNA extraction according to manufacturer’s instructions. Due to low 260/230 ratios (i.e., 1.0–1.6) following TRIzol extraction, all RNA samples were then re-extracted (phenol-chloroform) and precipitated following standard methods [31]. This was followed by DNaseI treatment and column purification using RNase-free DNase Set and RNeasy Mini Kit (QIAGEN). All procedures were conducted according to manufacturer instructions, and as described in Xue et al. [29]. RNA integrity was verified by 1% agarose gel electrophoresis, and RNA purity and quantity were assessed by NanoDrop UV spectrophotometry (NanoDrop, Thermo Scientific, Mississauga, ON, Canada). DNased and column-purified RNA samples had A260/280 and A260/230 ratios of 1.8–2.2. All cDNAs were synthesized by reverse transcription of 1 μg of DNaseI-treated, column-purified total RNA from each sample, with 1 µL of random primers (250 ng; Invitrogen), 1 µL of dNTPs (0.5 mM final concentration; Invitrogen), 4 µL of 5× first-strand buffer (1× final concentration; Invitrogen), 2 µL of DTT (10 mM final concentration; Invitrogen) and 1 µL of Moloney murine leukemia virus (M-MLV) reverse transcriptase (RT) (200 U; Invitrogen) at 37 °C for 50 min, following the manufacturer’s instructions, and as described in Xue et al. [29]. The total reaction volume was 20 μL. Finally, all cDNAs were diluted 40× with nuclease-free water (Invitrogen) prior to the qPCR.

### 2.4. Microarray Hybridization and Data Acquisition

Eight fish (2 from each of the 4 dietary tanks) from each of the 2 extreme ω6:ω3 treatments (high ω6 or high ω3) were used in the microarray analysis (i.e., 16 fish total), using a common reference design. Four array slides were used in the current study, and each array contained 2 fish per treatment, which were randomly selected. The common reference was made by an equal quantity of each DNase I-treated, column-purified total RNA liver sample. The microarray experiment was performed as described in Xue et al. [29]. Briefly, anti-sense amplified RNA (aRNA) was in vitro transcribed from 1 μg of each column-purified RNA or reference pooled RNA using Ambion’s Amino Allyl MessageAmp II aRNA Amplification kit (Life Technologies, Burlington, ON, Canada), following the manufacturer’s instructions. The quantity and quality of aRNA were assessed using NanoDrop spectrophotometry and 1% agarose gel electrophoresis, respectively. Then, 20 μg of each aRNA were precipitated overnight, following standard molecular biology procedures, and re-suspended in coupling buffer. Each individual aRNA sample was labeled with Cy5 (i.e., experimental samples), whereas the reference pool was labeled with Cy3 (i.e., common reference) fluor (GE HealthCare, Mississauga, ON, Canada), following the manufacturer’s instructions. The “microarray” function of the NanoDrop spectrophotometer was used to measure the labeling efficiency of the aRNA. The labeled aRNA (825 ng) from each experimental sample (i.e., Cy5) was mixed with an equal quantity of labeled aRNA from the common reference (i.e., Cy3), for each array, and the resulting pool was fragmented, following the manufacturer’s instructions (Agilent, Mississauga, ON, Canada). Each pool was co-hybridized to a consortium for Genomic Research on All Salmonids Project (cGRASP)-designed 4 × 44 K salmonid oligonucleotide microarray (GEO accession # GPL11299) [27] (Agilent). Finally, the arrays were hybridized at 65 °C for 17 h with rotation (10 rpm), using an Agilent hybridization oven. The microarray slides were washed immediately after hybridization as per the manufacturer’s instructions.

Each microarray slide was scanned at 5 μm resolution with 90% laser power using a ScanArray Gx Plus scanner and ScanExpress v4.0 software (Perkin Elmer, Waltham, MA, USA), and the Cy3 and Cy5 channel photomultiplier tube (PMT) settings were adjusted to balance the fluorescence signal between channels. The resulting raw data were saved as TIFF images, and the signal intensity data were extracted using Imagene 9.0 (BioDiscovery, El Segundo, CA, USA). Removal of low-quality or flagged spots on the microarray, as well as log_2_-transformation and Loess-normalization of the data, were performed using R and the Bioconductor package mArray [32]. Features absent in more than 25% (i.e., 4 out of 16 arrays) of the arrays were omitted, and the missing values were imputed using the EM_array method and the LSimpute package [33,34]. The final dataset used for statistical analyses consisted of 10,264 probes for all arrays (GEO accession number: GSE139418; https://www.ncbi.nlm.nih.gov/geo/query/acc.cgi?acc=GSE139418.

### 2.5. Microarray Data Analysis

The Significance Analysis of Microarrays (SAM) algorithm [35] was performed to identify genes that were significantly differentially expressed between the two extreme ω6:ω3 treatments. A false discovery rate (FDR) threshold of 10% was used with the Bioconductor package siggenes [36] in R. For the identification of additional transcripts that were differentially expressed between the two dietary treatments, the Rank Products (RP) method was also used, as this method is less sensitive to high biological variability [37,38]. The latter analysis was performed at a percentage of false-positives (PFP) threshold of 10%, using the Bioconductor package RankProd [39]. Due to high background signal in the first slide (i.e., slide # 11,502), no genes were initially identified as significantly differentially expressed; therefore, this slide was removed from the analyses. In order to maximize our capacity to identify differentially expressed genes, gene lists were obtained with 2 and 3 of the remaining slides (consisting of 4 and 6 fish per treatment, respectively). Each slide is composed of 4 arrays, i.e., 4 biological replicates analyzed per slide.

The resulting gene lists were annotated using the contiguous sequences (contigs) that were used for the design of the 60 mer oligonucleotide probes of the array [27]. Annotations were performed manually with a BLASTx alignment against the NCBI non-redundant (nr) amino acid database using an E-value threshold of 10^−5^. The best BLASTx hits corresponding to putative *Homo sapiens* orthologues were used to obtain gene ontology (GO) terms manually from the UniProt Knowledgebase (http://www.uniprot.org/, accessed on 4 November 2020).

### 2.6. qPCR Study and Data Analysis

Transcript expression levels of 10 genes of interest (GOI) (Table 1), identified as differentially expressed in the microarray analyses, were assayed by qPCR. In addition to the high ω6 and high ω3 treatments, the qPCR analysis also included liver samples from fish fed the balanced diet. In addition to the microarray-identified GOI, BLASTn searches using publicly available Atlantic salmon cDNA sequences (i.e., in NCBI’s non-redundant nucleotide (nt) and expressed sequence tags (EST) databases) were used to identify paralogues for each GOI, as described in Caballero-Solares et al. [40].

Paralogue-specific primers were used for *eif4b*, *htra1*, *lect2* and *helz2* (Table S3 and Figures S1–S4). The sequences of the primer pairs used in qPCR, GenBank accession number of sequences used for primer design, and other details are presented in Table 1. Notably, primers for the transcript *lhpl4* (GenBank accession number NM_001146670) failed quality testing, and thus, this transcript was not included in the qPCR study. In addition, the 60 mer microarray probe for *mtco1* (C188R069) is affiliated with a rainbow trout sequence, and had relatively low identity (i.e., <85%) with available *Salmo salar* sequences (using NCBI’s EST and nt databases) and, therefore, was excluded from the qPCR study. The program Primer 3 (http://frodo.wi.mit.edu, accessed on 19 October 2019) was used for primer design. Each primer pair was quality-tested, including standard curve and dissociation curve to ensure that a single product was amplified with no primer dimers [32,41]. Primer pairs were quality-tested using the 7500 Fast Real Time PCR system (Applied Biosystems/Life Technologies, Foster City, CA, USA). The amplification efficiency [42] of each primer pair was determined using a 5-point 1:3 dilution series starting with cDNA representing 10 ng of input total RNA. Two pools were generated (i.e., high ω3 pool and high ω6 pool), with each pool consisting of 8 fish (and each fish contributing an equal quantity to the pool). The reported primer pair amplification efficiencies are an average of the two pools, except if one pool showed poor efficiency or spacing (i.e., *p43*, *eif2a*, *htra1a*, *helz2a* and *helz2b*, where one pool was used due to low expression levels). A 5-point 1:2 dilution series was used for the primers *mtco2* and *helz2b* as these transcripts had lower expression levels (fluorescence threshold cycle (C_T_) values of ~30 and 31, respectively). Furthermore, amplicons were checked by 1.5% agarose gel electrophoresis and compared with the 1 kb plus DNA Ladder (Invitrogen) to ensure that the correct size fragment was amplified.

To select the most suitable normalizer genes, six candidate normalizers were tested based on our previous qPCR studies (*rpl32*, *actb*, *eef1α-1*, *eef1α-2*, *abcf2*, *pabpc1*) [15,29], and salmon literature on reference genes (*actb*, *eef1α-1*, *eef1α-2*) [43]. Their qPCR primers were quality-tested as described above. Then, their C_T_ values were measured using cDNA (corresponding to 5 ng of input total RNA) of 6 randomly selected fish per treatment (18 total). The geNorm algorithm [44] was used to analyze their expression stability. *Rpl32* and *eef1α-2* were shown to be the most stable (i.e., geNorm M-values of 0.30 and 0.25, respectively) among the 6 candidate reference genes and, therefore, were selected as normalizers.

All PCR amplifications were performed in a total reaction volume of 13 μL and consisted of 4 μL of cDNA (5 ng input total RNA), 50 nM each of forward and reverse primer and 1× Power SYBR Green PCR Master Mix (Applied Biosystems), and nuclease-free water (Invitrogen). The qPCR reactions, including no-template controls, were performed in technical triplicates using the ViiA 7 Real-Time PCR System (384-well format) (Applied Biosystems, Foster City, CA, USA) and the Power SYBR Green I dye chemistry. The Real-Time analysis program consisted of 1 cycle of 50 °C for 2 min, 1 cycle of 95 °C for 10 min, followed by 40 cycles (of 95 °C for 15 s and 60 °C for 1 min), with the fluorescence signal data collection after each 60 °C step. When a C_T_ value within a triplicate was greater than 0.5 cycles from the other two values, it was considered to be an outlier, discarded and the average C_T_ of the remaining two values was calculated. The relative quantity (RQ) of each transcript was calculated using ViiA 7 Software v1.2 (Applied Biosystems) for Comparative CT (ΔΔC_T_) analysis [45], with primer amplification efficiencies incorporated (Table 1). The expression levels of each GOI were normalized to both normalizer genes, and the sample with the lowest normalized expression was used as the calibrator sample (i.e., RQ = 1.0) for each GOI, as in [46]. Transcript expression data are presented as RQ values (mean ± SE) relative to the calibrator.

### 2.7. Statistical Analyses

#### 2.7.1. qPCR Data

A general linear model with tank nested in diet, followed by a Tukey pairwise comparison (*p* < 0.05), was used to identify significant differences among dietary treatments at week 12. In cases where significant tank effect was observed (*p* < 0.05), a one-way ANOVA followed by a Tukey pairwise comparison post-hoc test was performed (Minitab 17 Statistical Software, State College, PA, USA). The RQ data are presented as mean ± SE. Each dietary treatment group was tested for outliers using Grubb’s test (*p* < 0.05). In total, 9 RQ values were identified as statistical outliers in the entire dataset (i.e., out of 322 values), and excluded from the study. Each GOI had a minimum of 6 samples per dietary treatment, while most GOI had a sample size of 7–8 per dietary treatment. The qPCR fold-changes were calculated by dividing the mean RQ value of the high ω6 fish by that of the high ω3 fish. Finally, residuals were tested to verify normality, independence, and homogeneity of variance. Normality was examined using the Anderson–Darling test. If the test failed (*p* < 0.05), a one-way ANOVA on ranks was performed, which was followed by the Kruskal–Wallis test (SigmaPlot, Systat Software, Inc., Version 13, San Jose, CA, USA). In all cases, differences were considered statistically significant when *p* < 0.05.

#### 2.7.2. Correlation Analyses of qPCR and Lipid Composition Data

Tissue lipid composition (muscle and liver) and growth performance of salmon fed varying ω6:ω3 diets were previously published by Katan et al. [26]. Pearson correlation analyses were performed in the current study to identify the relationships between hepatic transcript expression (i.e., qPCR data), tissue composition (i.e., % FA and lipid classes), and growth parameters (i.e., weight gain (WG), condition factor (CF)), using individual fish. All GOI in the qPCR study were used in the correlation analysis in order to identify differences between the liver and muscle tissue. Only ω3 and ω6 FA that accounted for >0.5% of the total FA in the tissue (average of each treatment) were included in the analyses. Furthermore, hierarchical clustering was used to group transcripts and lipid composition (using group average in PRIMER (Version 6.1.15, Ivybridge, UK)). IBM SPSS Statistics was used for the correlation analyses.

## 3. Results

### 3.1. Liver Microarray Analysis

RP analysis detected nine differentially expressed features (PFP < 10%; Table 2). Eight of these features (i.e., *lhpl4*, *htra1b*, *mtco2*, *lect2a*, *rpl18*, *helz2a*, *itgb5*, and *mtco1*) were identified analyzing data from two slides (slides # 11,504–11,505; comprising four fish per treatment), and one (i.e., *mef2d*) was identified analyzing data from three slides (slides # 11,503–11,505; comprising six fish per treatment). Two features (i.e., *lhpl4* and *htra1b*) showed higher expression in the high ω6 fish (4.78- and 3.57-fold change, respectively), while the other seven RP-identified features (i.e., *mtco2*, *lect2a*, *rpl18*, *mef2d*, *helz2a*, *itgb5*, and *mtco1*) showed down-regulation in the high ω6 fish (fold-change ranged from −3.27 to −7.11).

SAM analysis identified *p43*, *eif2a*, *eif4b1*, and *itgb5* as differentially expressed genes (FDR < 10%) between the high ω6 and high ω3 fed salmon, using three slides (slides # 11,503–11,505) (Table 2). These genes were down-regulated in the high ω6 compared with the high ω3 fed fish (fold-change values ranged from −2.79 to −5.12). One feature (*itgb5*) was represented in both SAM and RP analysis, and was down-regulated in the high ω6 compared to the high ω3 fed fish, in both analyses (−5.12 and −5.25- fold-change, respectively).

Putative identities were determined for the 12 microarray-identified features, and functional annotations (i.e., GO terms) were collected for them (Table 2). The microarray-identified gene *lhpl4* (4.78-fold up-regulated) is involved in the nervous system, with GO annotations “regulation of inhibitory synapse assembly” and “GABA receptor binding”. The feature *htra1b* (3.57-fold up-regulated) was classified as a gene involved in cell proliferation and showed the functional annotations “positive regulation of epithelial cell proliferation”, “proteolysis” and “extracellular space” (Table 2). Several informative microarray features represented genes involved in translation, such as *p43*, *eif2a*, *eif4b1*, and *rpl18* (−2.79 to −4.37-fold down-regulated), with the associated GO terms “tRNA binding”, “aminoacyl-tRNA synthetase multienzyme complex”, “translational initiation”, “ribosome assembly”, and “structural constituent of ribosome”. Furthermore, the GO terms “defense response to virus”, “inflammatory response” and “response to wounding” were also identified with *p43*. The features *mtco2* and *mtco1* (−3.27- and −7.11-fold down-regulated, respectively) were classified as mitochondrion respiratory chain components, and showed the GO terms “electron transport chain”, “oxidation-reduction process” and “cytochrome-c oxidase activity”. Other microarray-identified features corresponded to immune- and inflammation-related genes such as *lect2a* (with the GO terms “chemotaxis” and “metal ion binding”) and *itgb5* (“antigen processing and presentation” and “phagocytic vesicle”), and showed down-regulation in the high ω6 fed fish (−3.48 to −5.25-fold-change, respectively). The gene *mef2d* (−4.54-fold down-regulated) is involved in muscle cell proliferation, and in neuronal cell differentiation and survival, with the associated GO terms “muscle organ development”, “skeletal muscle cell differentiation” “nervous system development”, “apoptotic process” and “DNA-binding transcription factor activity” (Table 2). Further, the microarray-identified feature *itgb5* was associated with the GO term “muscle contraction”. Finally, the gene *helz2a* (−4.71-fold down-regulated) was classified as a gene involved in lipid metabolism regulation by peroxisome proliferator-activated receptor alpha (PPARα), and showed the functional annotations “regulation of lipid metabolic process”, “nuclear receptor transcription activity”, “ATP binding”, “metal ion binding”, “hydrolase activity” and “ribonuclease activity”.

### 3.2. qPCR Study

Ten microarray-identified genes were used in the qPCR study. All genes, with the exception of *mtco2* and *rpl18*, showed an agreement in the direction of expression fold-change (i.e., up- or down-regulation) between the microarray and qPCR studies (Table 3). The microarray-identified *helz2a* showed significantly lower transcript expression in the high ω6 compared to the high ω3 fed fish (−1.49-fold; *p* = 0.04). The paralogue *helz2b* showed significantly lower expression in both the high ω6 and balanced groups compared to the high ω3 fed fish (−1.61-fold; *p* = 0.002). The transcript *mef2d* showed significantly lower expression in the high ω6 compared to the balanced fed fish (−1.27-fold; *p* = 0.03), and a lower expression trend in the high ω6 compared to the high ω3 fish (−1.22-fold; *p* = 0.06). Both paralogues of *htra1* were numerically higher (although not statistically significant) in the high ω6 compared to the balanced and high ω3 fish (1.34–1.57-fold and 3.75–2.09-fold; *p* = 0.25 and 0.07, respectively) (Table 3).

### 3.3. Correlations between Hepatic qPCR Transcript Expression and Liver Lipid Composition

**Figure 1:**
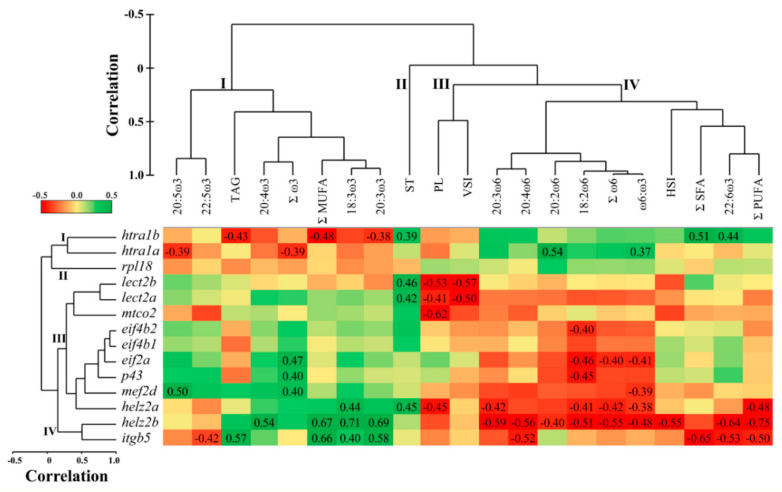
Pearson correlation matrix and hierarchical clustering of liver transcript expression (qPCR relative quantity values (RQ)), liver lipid composition, and somatic indices in Atlantic salmon fed diets with varying ω6 to ω3 fatty acid ratios. Correlation coefficients were described when correlations were statistically significant (*p* < 0.05). Red signifies negative and green signifies positive relationships. ΣSFA, ΣMUFA, and ΣPUFA represents total saturated, monounsaturated and polyunsaturated fatty acids, respectively. 20:5ω3, 22:6ω3, and 20:4ω6 represent EPA, DHA, and ARA, respectively. TAG, ST and PL represent triacylglycerols, sterols, and phospholipids, respectively. HSI and VSI represent hepatosomatic and viscerosomatic indices, respectively.

**Figure 2:**
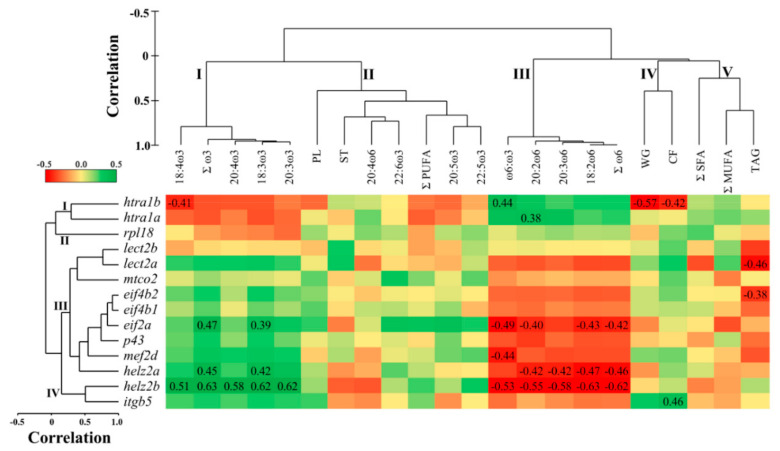
Pearson correlation matrix and hierarchical clustering of liver transcript expression (qPCR relative quantity values (RQ)), muscle lipid composition, and growth in Atlantic salmon fed diets with varying ω6 to ω3 fatty acid ratios. Correlation coefficients were described when correlations were statistically significant (*p* < 0.05). Red signifies negative and green signifies positive relationships. ΣSFA, ΣMUFA, and ΣPUFA represent total saturated, monounsaturated and polyunsaturated fatty acids, respectively. 20:5ω3, 22:6ω3, and 20:4ω6 represent EPA, DHA, and ARA, respectively. TAG, ST and PL represent triacylglycerols, sterols, and phospholipids, respectively. WG and CF represent weight gain and condition factor, respectively.

Hierarchical clustering of the qPCR transcripts showed four separate clusters (Figure 1 and Figure 2). The first cluster consisted of both paralogues of *htra1*. The second cluster comprised *rpl18* only. The third cluster included some of the immune- and inflammation-related transcripts such as *lect2*, *p43*, and *helz2a*, as well as *mef2d*, *eif2a*, *eif4b*, and *mtco2*. The transcripts *helz2b* and *itgb5* composed the fourth cluster.

Cluster analysis of liver lipid composition and somatic indices showed four clusters (Figure 1). Cluster one consisted of the ω3 FA: 18:3ω3, 20:3ω3, 20:4ω3, 20:5ω3, 22:5ω3, as well as the sums of ω3 (Σω3) and monounsaturated FA (ΣMUFA), and the lipid class triacylglycerols (TAG). The lipid class sterols (ST) represented cluster two, while total phospholipids (PL) segregated with viscerosomatic index (VSI) in cluster three. Cluster four consisted of the ω6 FA: 18:2ω6, 20:2ω6, 20:3ω6, 20:4ω6, Σω6, and the ratio ω6:ω3; in addition, 22:6ω3, the sums of PUFA (ΣPUFA) and saturated fatty acids (ΣSFA), and the hepatosomatic index (HSI) were associated with this cluster.

The hepatic transcript expression of *htra1b* was negatively correlated with TAG, ΣMUFA, and 20:3ω3, and positively with ST, ΣSFA, and 22:6ω3 (*p* = 0.009–0.043; Figure 1). *Htra1a* showed negative correlations with 20:5ω3 and Σω3, and positive with 20:2ω6, and ω6:ω3 (*p* = 0.006–0.047). Both paralogues of *lect2* were correlated negatively with PL and VSI, and positively with ST (*p* = 0.004–0.032). Transcript expression of *mtco2* was negatively correlated with PL (*p* = 0.001), while that of *eif4b2* was negatively correlated with 18:2ω6 (*p* = 0.036; Figure 1). Both *eif2a* and *p43* transcript expression correlated negatively with 18:2ω6 (*p* = 0.019 and 0.021, respectively) and positively with Σω3, whereas *eif2a* alone correlated negatively with Σω6 and ω6:ω3 (*p* = 0.037 and 0.033, respectively). *Mef2d* was correlated negatively with ω6:ω3 and positively with 20:5ω3 (*p* = 0.042, and 0.011, respectively), while the three transcripts *eif2a*, *p43*, and *mef2d* showed positive correlations with Σω3 (*p* = 0.016–0.037). Furthermore, both paralogues of *helz2* correlated negatively with ω6 PUFA (i.e., 18:2ω6, 20:3ω6, Σω6), ω6:ω3, and ΣPUFA, and positively with 18:3ω3 (*p* = 0.0001–0.047). However, *helz2b* had negative correlations with additional ω6 (i.e., 20:2ω6, 20:4ω6; *p* = 0.038 and 0.004, respectively), and positive correlations with ω3 PUFA (i.e., 20:3ω3, 20:4ω3; *p* = 0.0001 and 0.005, respectively). In addition, *helz2b* correlated negatively with 22:6ω3 and HSI, and positively with ΣMUFA (*p* = 0.0001–0.005). In contrast, *helz2a* correlated negatively with PL, and positively with ST (*p* = 0.021). Finally, *itgb5* had negative correlations with 22:5ω3, 20:4ω6, ΣSFA, 22:6ω3, and ΣPUFA, and positive correlations with TAG, ΣMUFA, 18:3ω3, and 20:3ω3 (*p* =0.001–0.034; Figure 1).

### 3.4. Correlations between Hepatic qPCR Transcript Expression and Muscle Lipid Composition

Muscle tissue lipid composition and growth showed five separate clusters (Figure 2). Cluster one consisted of the ω3 FA: 18:3ω3, 18:4ω3, 20:3ω3, 20:4ω3, and Σω3. Cluster two included ΣPUFA, and the LC-PUFA: 20:5ω3, 22:5ω3. 22:6ω3, and 20:4ω6. Furthermore, the lipid classes ST and PL were grouped in cluster two. Cluster three grouped the ω6 FA: 18:2ω6, 20:2ω6, 20:3ω6, as well as Σω6 and the ratio ω6:ω3. Cluster four showed the growth parameters (i.e., WT and CF), while cluster five grouped TAG, ΣSFA and ΣMUFA.

The hepatic transcript expression of *htra1b* was correlated negatively with muscle 18:4ω3 and growth parameters WG and CF, and positively with ω6:ω3 (*p* = 0.004–0.037), while that of *htra1a* showed positive correlation with 20:2ω6 (*p* = 0.049; Figure 2). The transcript expression of *lect2a* and *eif4b2* was negatively correlated with TAG (*p* = 0.021 and 0.049, respectively). *Eif2a* was correlated negatively with muscle ω6 FA (i.e., 18:2ω6, 20:2ω6, Σω6) and ω6:ω3, and positively with ω3 FA (18:3ω3 and Σω3) (*p* = 0.014–0.045; Figure 2). *Mef2d* was negatively correlated with ω6:ω3 (*p* = 0.026). Further, both paralogues of *helz2* were negatively correlated with ω6 FA (18:2ω6, 20:2ω6, 20:3ω6, Σω6), and positively correlated with ω3 (i.e., 18:3ω3 and Σω3) (*p* = 0.001–0.032). *Helz2b* alone correlated negatively with ω6:ω3, and positively with 18:4ω3, 20:3ω3, and 20:4ω3 (*p* = 0.002–0.01). Finally, *itgb5* showed a positive correlation with CF (*p* = 0.024).

### 3.5. Overlapping Lipid–Gene Correlations between the Liver and Muscle Analyses

**Figure 3:**
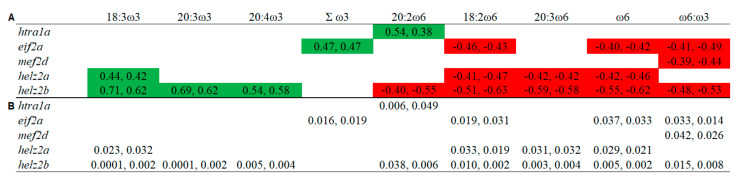
Overlapping Pearson correlations between the liver and muscle analyses. Liver transcript expression (qPCR relative quantity values (RQ)) of GOIs was correlated with liver and muscle lipid composition in Atlantic salmon fed diets with varying ω6 to ω3 fatty acid ratios. Statistically significant (*p* < 0.05) correlations are shown. Green and red cells signify positive and negative relationships, respectively. Upper panel shows correlation coefficients (**A**), and lower panel depicts *p*-values (**B**). Commas separated values from the liver and muscle analyses, respectively (**A**,**B**).

Some significant correlations showed an overlap between the liver and muscle analyses (Figure 3). The hepatic transcript expression of *htra1a* was correlated positively with 20:2ω6 in both tissues. The expression of *eif2a* was correlated positively with Σω3, and negatively with 18:2ω6, Σω6 and ω6:ω3, while that of *mef2d* showed negative correlations with ω6:ω3 in both liver and muscle. The transcript expression of *helz2a* was correlated positively with 18:3ω3, and negatively with 18:2ω6, 20:3ω6 and Σω6, while that of *helz2b* correlated positively with 18:3ω3, 20:3ω3 and 20:4ω3, and negatively with 18:2ω6, 20:2ω6, 20:3ω6, Σω6 and ω6:ω3 (Figure 3). The hepatic transcript expression of most genes (all except *helz2b* with 20:4ω3) showed stronger positive correlations with liver compared to muscle FA. However, negative correlations were mostly (all except *eif2a* with 18:2ω6, and both *helz2* paralogues with 20:3ω6) more significant with muscle compared with the liver FA (Figure 3).

## 4. Discussion

The microarray study indicated that dietary variation in ω6:ω3 resulted only in small changes in the liver transcriptome of salmon fed plant-based diets. This can partly be explained by the fact that growth performance and somatic indices were not significantly affected by diet [26]. It was previously shown that different replacements of FO with camelina oil had no impact on Atlantic cod (*Gadus morhua*) growth, and resulted in only one microarray-identified gene that showed a significant difference in spleen basal expression between treatments [47]. Furthermore, our results are in line with previous microarray studies, which demonstrated that dietary replacement of fish meal (FM) and FO with terrestrial ingredients resulted in subtle gene expression changes in Atlantic salmon distal intestine [48], head kidney [49], and liver [50]. However, Atlantic salmon fed soy and linseed oils showed large alterations in hepatic gene expression compared to those fed FO [51]. Differences in the numbers of responsive transcripts between Leaver et al. [51] and the current study could be related to dietary lipid sources and studied time points.

Several transcripts that play important roles in immune and inflammatory response (*lect2a*, *itgb5*, *helz2a*, *p43*), lipid metabolism (*helz2a*), cell proliferation (*htra1b*), muscle and neuronal cell development (*mef2d*), and translation (*eif2a*, *eif4b1*, *p43*) were identified by our microarray study as diet-responsive. All transcripts, with the exception of *mtco2* and *rpl18*, showed an agreement in the direction of expression fold-change between the microarray and the qPCR analyses (Table 3). The 60mer microarray probe representing the transcript *mtco2*, which was designed using a rainbow trout cDNA sequence, showed only 87% similarity (see Materials and Methods) with the *Salmo salar* cDNA sequence that was used in the qPCR study (and other *S. salar* sequences in NCBI databases). This fact, as well as other limitations (e.g., mRNA regions targeted by the qPCR primers and microarray probe may not be the same; possibility of contig misassembly) could have contributed to the disagreement between microarray and qPCR results [32].

Hepatic *helz2a* showed a significant differential expression between the high ω6 and high ω3 fed fish in the microarray experiment, and both paralogues of this transcript (i.e., *helz2a* and *helz2b*) were significantly down-regulated in the high ω6 compared to the high ω3 fed fish in the qPCR analysis. Interestingly, the transcript expression of *helz2b* was positively correlated with ω3 (i.e., 18:3ω3, 20:3ω3, 20:4ω3), and negatively with ω6 PUFA (i.e., 18:2ω6, 20:2ω6, 20:3ω6, 20:4ω6), Σω6 and ω6:ω3, in the liver tissue (Figure 1). In the muscle tissue, these PUFA (with the exception of 20:4ω6) were also correlated with hepatic *helz2b* expression (Figure 2). These data suggest that *helz2* is a potential novel molecular biomarker of tissue variation in ω6:ω3. The protein encoded by this gene is a nuclear transcriptional co-activator for PPARα [52,53,54], which is a master regulator of numerous genes involved in lipid metabolism processes (e.g., FA oxidation, and metabolism of bile acids, triacylglycerols, and retinoids) [55]. Additionally, HELZ2 was shown to have an antiviral function in mammals [56,57], and its gene was identified as an ancestral (between mammals and fish) interferon stimulated gene (ISG) with conserved components of antiviral immunity [58]. *Helz2* transcripts (referred to as VHSV-induced protein (*vig1*)) showed up-regulation in the head kidney of Atlantic salmon exposed to the viral mimic polyriboinosinic polyribocytidylic acid (pIC) [15]. The negative correlation between liver *helz2b* and HSI is not surprising, given the involvement of PPARα in hepatic FA β-oxidation, and in liver steatosis [59,60]. In addition, the observed positive correlations with ω3 PUFA are in line with the anti-inflammatory properties of PPARα [60]. In our previous study [26], it was observed that the fatty acid binding protein-encoding transcript *fabp10* showed an upregulation trend (*p* = 0.06) in the high ω3 compared to the balanced and high ω6 fed fish. Thus, this suggests that the high ω3 diet may have influenced the transport of ω3 FA in liver cells, and played a role in the activation of PPARα. The interaction between liver fatty acid transport and PPARα activation has been shown in previous mammalian studies [61,62]. Another potential mechanism that could explain the positive correlation between *helz2b* expression with ω3 PUFA, is that ω3 PUFA bind to PPARα with higher affinity than ω6 PUFA [63]. However, there is still a lack of knowledge about the interaction between dietary ω3 and ω6 PUFA, and the mechanisms by which they regulate PPARα activators [64].

*Mef2d* was identified in the microarray as down-regulated by the high ω6 diet, with qPCR showing significantly lower expression in the high ω6 compared to the balanced diet fed fish. Furthermore, hepatic *mef2d* expression was correlated positively with liver 20:5ω3 and Σω3, and negatively with the ratio of ω6:ω3 in both liver and muscle tissues. The gene *mef2*, characterized in zebrafish (*Danio rerio*) [65] and common carp (*Cyprinus carpio*) [66], is involved in skeletal and cardiac muscle development and differentiation, as well as in neuronal cell development [67,68,69,70]. Wei et al. [71] reported a significant increase in skeletal muscle *mef2c* transcript expression in pigs fed with linseed-enriched (10%) as compared with a control diet (0%). Additionally, a study with Atlantic salmon revealed that feeding with a synthetic FA (i.e., tetradecylthioacetic acid (0.25%) compared to a control diet (0%) increased the cardiosomatic index, and the cardiac expression of *mef2c* [72]. In relation to the liver, previous studies showed that members of the MEF2 family regulate the activation of hepatic stellate cells –a type of cell involved in liver fibrosis– in mice [73] and rats [74], and ω3 PUFA inhibited the proliferation and activation of these cells in mouse liver [75]. Further, Wang et al. [74] reported that *mef2d* was induced during hepatic stellate cells activation.These data may support the idea that the transcript *mef2* is responding to dietary FA (particularly ω3 PUFA) in vertebrates. However, as most studies examined the role of *mef2* expression in skeletal [71,76] and cardiac [72,77] muscle development, the interactions between *mef2d* and liver physiology are less understood in fish. Future studies should investigate the influence of dietary ω6:ω3 on liver *mef2d* expression, and their interaction with hepatic stellate cells in fish.

Serine protease HTRA1-encoding transcript (*htra1b*) was up-regulated in the high ω6 compared to the high ω3 fed fish, in the microarray study. *Htra1b* showed a similar trend of higher expression in the high ω6 fed fish (*p* = 0.07) in the qPCR analysis, and a similar fold-change (i.e., high ω6/high ω3) in the microarray and qPCR studies (Table 3). Further, hepatic *htra1b* was positively correlated with ΣSFA, 22:6ω3 and ST, and negatively with ΣMUFA, 20:3ω3 and TAG in the liver, while, in the muscle, it showed positive and negative correlations with ω6:ω3 and 18:4ω3, respectively. The transcript *htra1a* was positively correlated with 20:2ω6 and ω6:ω3, and negatively with ω3 PUFA (i.e., 20:5ω3 and Σω3) in the liver, and showed positive correlation with 20:2ω6 in the muscle. Serine protease HTRA1 function was linked to cell growth and apoptosis, as well as immune and inflammatory responses (by inhibiting the TGF-beta pathway) in mammalian tissues (e.g., eye, bone and liver) [78,79,80,81]. It was previously shown that dietary FM replacement with terrestrial plant alternatives induced higher hepatic *htra1* transcript expression in Atlantic salmon [82]. Conversely, replacing both dietary FM and FO by terrestrial plant alternatives down-regulated the transcription of *htra1a* and *htra1b* in Atlantic salmon liver [40]. Discrepancies between Caballero-Solares et al. [40] and the present study extend to the FA–transcript correlation analyses; while, in the present study, the transcript expression of *htra1* paralogues correlated positively and negatively with tissue ω6 and ω3 FA levels, respectively, the opposite tendency was observed in Caballero-Solares et al. [40]. However, unlike the previous studies [40,82], our study tested different mixes of vegetable oils while keeping FM/FO inclusion levels equal across diets. Therefore, although the studies cannot be directly compared, such discrepancies suggest that the regulation of HTRA1-mediated processes in the liver of Atlantic salmon depends on the combination of protein and lipid sources included in the diet. Finally the negative correlation observed between *htra1b* and growth parameters (i.e., WG and CF) in the current study (Figure 2) is interesting, as mammalian HTRA1 was negatively linked to skeletal muscle development and bone formation [80,83,84].

The immune related microarray features *lect2a* and *itgb5* were down-regulated in the high ω6 compared to the high ω3 fed fish, and an agreement was observed in the direction of expression fold-change between the microarray and qPCR studies. LECT2 is a multifunctional protein that plays a role in cell growth, neutrophil chemotactic activity, and innate immune response against pathogens in fish [85,86,87,88]. LECT2 also functions as a hepatokine that modulates the inflammatory response in mammals [89,90]. Earlier microarray studies reported down-regulation of hepatic *lect2* in Atlantic salmon fed terrestrial as compared with marine diets [29,40]. This may indicate that pro-inflammatory plant-based diets suppress the constitutive transcript expression of hepatic *lect2*. The observed negative correlation between both paralogues of *lect2* and VSI suggests that *lect2* suppression is related to higher lipid deposition. However, the interaction between fat deposition and immune response is very complex, and requires further investigation in fish [90,91]. Additionally, we showed a positive correlation between the transcript expression of both paralogues of *lect2* and liver sterol content. Interestingly, a previous study reported down-regulation of hepatic *lect2* in Atlantic salmon fed a cholesterol-supplemented diet as compared with a non-supplemented plant-based diet, and this coincided with reduced plasma phytosterols (i.e., sitosterol and campesterol) [92]. Although dietary sterol levels were not significantly different in our study, liver sterol concentration did vary among treatments [26]. Clearly, more studies are required in order to elucidate the impact of dietary and tissue cholesterol and phytosterols on the constitutive transcript expression of *lect2* in fish. Finally, the correlations observed in our study between *itgb5* and liver FA are in line with the notion that FA can regulate the mRNA and protein levels of integrins and other adhesion proteins in leukocytes and endothelial cells [93,94]. Interestingly, European seabass (*Dicentrarchus labrax*) fed a plant-based diet showed a down-regulation in hepatic *Integrin beta-2* compared to those fed a marine diet [95], while a reduction in the ω6:ω3 ratio of human lung cancer cells resulted in a delayed adhesion, and down-regulation of *integrin-α2* [96]. Taken together, these data suggest that the transcript expression of integrins may be impacted by dietary or tissue ω6:ω3.

Similar to *lect2a* and *itgb5*, the transcripts *p43*, *eif2a*, and *eif4b1* were down-regulated in the high ω6 fed fish in the microarray experiment, and they showed an agreement in the direction of dietary modulation with the qPCR study. The transcript expression of *p43*, *eif2a*, and *eif4b2* was negatively correlated with 18:2ω6, and both *p43* and *eif2a* were positively correlated with liver Σω3. Further, *eif2a* expression was positively correlated with 18:3ω3 and Σω3, and negatively correlated with 18:2ω6, 20:2ω6, Σω6 and ω6:ω3 in the muscle. The protein p43 is associated with a multi-tRNA synthetase complex, and regulates tRNA channeling in mammals [97]. In addition, p43 also encodes an apoptosis-induced cytokine, which regulates inflammation, wound healing, and angiogenesis [98,99,100]. Phosphorylation of the protein eIF4B was shown to stimulate translation in zebrafish [101,102] and yeast [103]. However, phosphorylation of eIF2A repressed translation in response to accumulation of misfolded proteins in the ER of several fish species [104,105,106]. Thus, changes in the expression patterns of *p43*, *eif2a* and *eif4b*, and the correlations observed with tissue lipid composition, suggest that some aspects of protein synthesis were influenced by dietary and tissue ω6:ω3. Previous mammalian studies demonstrated that translation is inhibited in apoptotic cells, and this was correlated with enhanced cleavage of the eukaryotic translation initiation factors eIF4B, eIF2, and others [107,108]. Thus, the fact that the high ω6 fed fish showed up-regulation of *htra1b* (Table 2) may suggest that apoptosis was associated with the observed modulation of translation-related transcripts (Table 2), and their correlations with tissue FA (Figure 1 and Figure 2). However, as our microarray study did not identify other well-known apoptosis biomarkers (e.g., genes encoding caspases and Bcl-2 family members), this can only be postulated. Further, the stimulatory effects of ω3 PUFA on protein synthesis [109] could be another potential mechanism explaining the positive correlations observed between *p43*, *eif2a*, and liver ω3 FA. Research examining the impact of replacing FO/FM with plant-based diets on protein synthesis in salmonids has been contradictory. Some authors showed an induction [82], while others showed a suppression [110] of these and other translation-related transcripts. Indeed, protein synthesis regulation in fish is a dynamic process, and is influenced by dietary formulations, genetic [50] and abiotic factors, protein requirement, growth, and the tissues examined [111,112].

## 5. Conclusions

Our 44 K microarray study demonstrated that high ω6 and high ω3 plant-based diets with varying ratios of ω6:ω3 (i.e., 2.7 and 0.4, respectively) resulted in a relatively low number of differentially expressed transcripts in salmon liver. However, the identified transcripts and/or their functional annotations suggested important roles in lipid metabolism (*helz2a*), cell proliferation (*htra1b*), immune and inflammatory response (*lect2a*, *itgb5*, *helz2a*, *p43*), control of muscle and neuronal cell development (*mef2d*), and translation (*eif2a*, *eif4b1*, *p43*). Two paralogues of *helz2* were down-regulated in the high ω6 compared to the high ω3 fed fish in the qPCR study. Significant positive correlations were observed between the hepatic transcript expression of *helz2b* and ω3 PUFA, while negative correlations were identified with ω6 PUFA and ω6:ω3, in both the liver and muscle tissues. This indicated that the PPARα activation-related transcript *helz2* is a potential novel molecular biomarker of tissue variation in ω6:ω3. Given these data and the importance of *helz2* as an ancestral vertebrate interferon stimulated gene, future studies should investigate the dietary ω6:ω3 impact on Atlantic salmon anti-viral response. The transcript *mef2d* was suppressed in the high ω6 compared to the balanced fed fish, and was negatively correlated with ω6:ω3 in both tissues. Our microarray study revealed that the upregulation of hepatic *htra1b* concurred with the suppression of immune- and inflammatory-related transcripts (i.e., *lect2a*, *p43*, *helz2a*, *helz2b*, and *itgb5*). This supported the idea proposed by other researchers [40,82] of a link between the dietary modulation of *htra1* and that of immune-related transcripts. Finally, the transcripts *p43*, *eif2a*, and *eif4b1* were significantly down-regulated in the high ω6 compared to the high ω3 fed fish in the microarray, and showed an agreement in the direction of expression fold-change between the microarray and qPCR studies. These data, along with the significant correlations observed between *p43*, *eif2a* and *eif4b2* expression and tissue PUFA, suggested that the molecular regulation of protein synthesis in the liver may have been impacted by dietary ω6:ω3.

## Figures and Tables

**Table 1:** qPCR primers.

Gene Name (Symbol) ^a^	Nucleotide Sequence (5′-3′) ^b^	Amplification Efficiency (%)	Amplicon Size (bp)	GenBank Accession Number
Serine protease HTRA1 a (*htra1a*) ^c^	F:GCTGATGTGGTGGAGGAGAT	113.3	127	NM001141717
	R:TCAAGCCGTCCTCTGACAC	-	-	-
Serine protease HTRA1 b (*htra1b*) ^c^	F:ATGATGACTCTCACACCAATGC	95.4	104	EG831192
	R:GTTTTTGGGATGACCTCGATT	-	-	-
Aminoacyl tRNA synthase complex-interacting multifunctional protein 1 (*p43*)	F:GGAAGACGAATGCAGAGGAC	97.2	82.0	BT044000
	R:GGAGCGGTCATTCACACTTT	-	-	-
Eukaryotic translation initiation factor 2A (*eif2a*)	F:TAAACCCAGATGCCCTTGAG	94.9	143	NM001140088
	R:GGCTTTCAGCTCGTCGATAG	-	-	-
Eukaryotic translation initiation factor 4B 1 (*eif4b1*)	F:CGCAGGGACCGGGATGAT	85.3	123	BT072661
	R:TCGGTCCTC5CTGTCCGC	-	-	-
Eukaryotic translation initiation factor 4B 2 (*eif4b2*)	F:CACATCCAGGAAGTACCTCT	87.4	94.0	DY739566
	R:TCGTCCTCCTTACCGCTGA	-	-	-
Cytochrome c oxidase subunit 2 (*mtco2*) ^d^	F:CACCGATTACGAAGACTTAGGC	107.9	136	DW554935
	R:TGAAACTAGGACCCGGATTG	-	-	-
Leukocyte cell-derived chemotaxin 2 precursor a (*lect2a*) ^c^	F:CAGATGGGGACAAGGACACT	94.6	150	BT059281
	R:GCCTTCTTCGGGTCTGTGTA	-	-	-
Leukocyte cell-derived chemotaxin 2 precursor b (*lect2b*) ^c^	F:ACAACTGGGGACAAGGACAG	84.8	125	DV106130
	R:CACTTTGCCGTTGAGTTTCA	-	-	-
60S ribosomal protein L18 (*rpl18*)	F:AGTTCCACGACTCGAAGATC	93.8	143	DW535031
	R:TTTTATTGTGCCGCACAAGGT	-	-	-
Myocyte-specific enhancer factor 2D (*mef2d*)	F:GCAGCAACATCAACAACAGC	89.5	160	XM014177143
	R:CTCATCTCTACCCAAGAGGA	-	-	-
Helicase with zinc finger domain 2 a (*helz2a*, alias *pric285a*) ^e^	F:GCAAGGTTGGGTATGAGGAA	91.3	149	BT072427
	R:TTCGGAGTTGCTCCAGTCTT	-	-	-
Helicase with zinc finger domain 2 b (*helz2b*, alias *pric285b*) ^e^	F:AGACGTAGTGGTTCGGATCG	82.0	145	EG928625
	R:GACCGTGATTTCGTCCAGTT	-	-	-
Integrin beta-5-like (*itgb5*) ^f^	F:CCTGCCAGCGGCTATGCAA	94.1	147	DW540995/ XM014165323
	R:AGGACTGACATGCCGTTGG	-	-	
Elongation factor 1 alpha-2 (*eef1α-2*) ^g^	F:GCACAGTAACACCGAAACGA	86.4	132	BG933853
	R:ATGCCTCCGCACTTGTAGAT	-	-	-
60S ribosomal protein 32 (*rpl32*) ^g^	F:AGGCGGTTTAAGGGTCAGAT	96.1	119	BT043656
	R:TCGAGCTCCTTGATGTTGTG	-	-	-

^a^ Bolded gene symbols refer to microarray-identified transcripts. ^b^ F is forward and R is reverse primer. ^c^ Primers that were previously published in Caballero-Solares et al. [40]. ^d^ The *Salmo salar* sequence of *mtco2* used in the qPCR assay showed 87% identity with the 60 mer microarray probe (C060R108) affiliated with rainbow trout (*Oncorhynchus mykiss*). ^e^ Primers that were previously published in Caballero-Solares et al. [15] (annotated as VHSV-induced protein in that study). Alias *pric285* stands for peroxisomal proliferator-activated receptor A interacting complex 285. ^f^ The Atlantic salmon sequences of *itgb5* used in the qPCR assay showed 86% identity with the 60mer microarray probe (C002R106) affiliated with rainbow trout. Primers were designed based on common regions between DW540995 and XM014165323. ^g^ Primers that were previously published in Katan et al. [26].

**Table 2:** Microarray-identified transcripts that were significantly differentially expressed in the liver of salmon fed high ω6 compared to high ω3 diet.

Probe ID ^a^	BLASTx Identification ^b^				Gene Ontology (GO) of Putative Human Orthologues ^d^	Fold-Change ^e^
	Best Named BLASTx Hit (Species) ^c^	Accession No.	E-Value	% ID (AA)		
C187R103	Lipoma HMGIC fusion partner-like 4 protein (*lhpl4*) (*Salmo salar*)	NP_001140142	0	272/272 (100%)	BP: regulation of inhibitory synapse assembly, gamma-aminobutyric acid receptor clustering. MF: protein binding, GABA receptor binding. CC: inhibitory synapse, postsynaptic membrane, cell projection, plasma membrane, cell junction.	4.78
C231R170	Serine protease HTRA1 (*htra1b*) (*Salvelinus alpinus*)	XP_023864611	4e−171	248/256 (97%)	BP: proteolysis, extracellular matrix disassembly, negative regulation of transforming growth factor beta receptor signaling pathway, negative regulation of defense response to virus, positive regulation of epithelial cell proliferation. MF: serine-type endopeptidase and peptidase activity, insulin-like growth factor binding, hydrolase activity. CC: collagen-containing extracellular matrix, extracellular space, plasma membrane, cytoplasm.	3.57
C103R052	Aminoacyl tRNA synthase complex-interacting multifunctional protein 1 (*p43*) (*Salmo trutta*)	XP_029622221	0	321/326 (98%)	BP: inflammatory response, apoptotic process, response to wounding, tRNA aminoacylation for protein translation, defense response to virus, leukocyte migration, angiogenesis, chemotaxis, positive regulation of glucagon secretion. MF: RNA binding, tRNA binding, protein binding, cytokine activity, protein homodimerization activity. CC: aminoacyl-tRNA synthetase multienzyme complex, nucleus, cytosol, endoplasmic reticulum, extracellular region.	−2.79
C067R040	Eukaryotic translation initiation factor 2A (*eif2a*) (*Salmo salar*)	NP_001133560	0	576/576 (100%)	BP: translational initiation, ribosome assembly, protein phosphorylation, SREBP signaling pathway, response to amino acid starvation. MF: translation initiation factor activity, cadherin binding, ribosome binding, tRNA binding, protein binding. CC: blood microparticle, extracellular space, cytosolic small ribosomal subunit.	−3.13
C253R093	Eukaryotic translation initiation factor 4B (*eif4b1*) (*Salvelinus alpinus*)	XP_023852969	6e−11	37/40 (93%)	BP: translational initiation, eukaryotic translation initiation factor 4F complex assembly. MF: RNA binding, protein binding, translation initiation factor activity, RNA strand annealing activity. CC: polysome, cytosol, eukaryotic translation initiation factor 4F complex.	−3.23
C060R108	Cytochrome c oxidase subunit 2 (*mtco2*) (*Oncorhynchus masou masou*)	ASB29545	7e−74	115/182 (63%)	BP: electron transport chain, oxidation-reduction process. MF: cytochrome-c oxidase activity, copper ion binding, metal ion binding, oxidoreductase activity. CC: membrane, respirasome, mitochondrion.	−3.27
C159R112	Leukocyte cell-derived chemotaxin 2 precursor (*lect2a*) (*Salmo salar*)	ACI67916	6e−102	155/156 (99%)	BP: chemotaxis, skeletal system development. MF: protein binding, metal ion binding. CC: cytoplasm, extracellular space.	−3.48
C152R057	60S ribosomal protein L18 (*rpl18*) (*Salmo trutta*)	XP_029599741	3e−122	172/173 (99%)	BP: translation, viral transcription, SRP-dependent cotranslational protein targeting to membrane. MF: structural constituent of ribosome, protein binding, RNA binding. CC: ribosome, cytosolic large ribosomal subunit, cytosol.	−4.37
C133R018	Myocyte-specific enhancer factor 2D (*mef2d*) (*Oncorhynchus mykiss*)	XP_021427816	3e−70	193/193 (100%)	BP: positive regulation of vascular smooth muscle cell proliferation, muscle organ development, skeletal muscle and neuronal cell differentiation, apoptotic process, positive regulation of transcription by RNA polymerase II, adult heart development, nervous system development. MF: DNA-binding transcription factor activity, RNA polymerase II-specific, protein binding, histone deacetylase binding, protein heterodimerization activity. CC: nucleus, nuclear chromatin, intracellular membrane-bounded organelle, nucleoplasm.	−4.54
C065R088	Helicase with zinc finger domain 2 (*helz2a* alias, *pric285a*) (*Salmo trutta*)	XP_029548942	0	694/714 (97%)	BP: regulation of lipid metabolic process, positive regulation of transcription by RNA polymerase II, nuclear-transcribed mRNA catabolic process, nonsense-mediated decay. MF: nuclear receptor transcription activity, helicase activity, ribonuclease activity, hydrolase activity, RNA binding, ATP binding, protein binding, metal ion binding. CC: nucleus, membrane, nucleoplasm.	−4.71
C002R106 *	Integrin beta-5-like (*itgb5*) (*Oncorhynchus mykiss*)	XP_021453113	0	283/315 (90%)	BP: cell adhesion mediated by integrin, integrin-mediated signaling pathway, muscle contraction, antigen processing and presentation of exogenous peptide antigen via MHC class I, TAP-dependent, viral process, transforming growth factor beta receptor signaling pathway. MF: protein binding, signaling receptor activity, virus receptor activity. CC: cell surface, extracellular exosome, phagocytic vesicle, plasma membrane, integrin complex.	−5.12
C188R069	Cytochrome c oxidase subunit 1 (*mtco1*) (*Oncorhynchus tshawytscha*) *	NP_148940	0	410/437 (94%)	BP: oxidation-reduction process, oxidative phosphorylation, electron transport chain, aerobic respiration. MF: oxidoreductase activity, cytochrome-c oxidase activity, heme binding, metal ion binding. CC: mitochondrial inner membrane, respiratory chain complex IV, respirasome.	−7.11

^a^ Refers to the identity of the probe on the 44 K array. Probes that are shown in bold font are features that were identified by SAM (FDR < 10%), and the remaining features were identified by RP analysis (PFP < 10%). The probe with an asterisk represents a feature that was identified in both SAM and RP analysis. Two 4 × 44 K array slides (slides # 11,504–11,505; representing 4 fish per treatment) were used in the RP analysis. However, the RP-identified *mef2d* was obtained using 3 slides (slides # 11,503–11,505; representing 6 fish per treatment). SAM-identified features were obtained using three slides (slides # 11,503–11,505). ^b^ Genes were identified by BLASTx, using the contig from which the microarray probe was designed against the NCBI non-redundant database. The best BLASTx hit with E-value < 10^−5^ and an informative protein name was used, and presented with species name, GenBank accession number, E-value and % amino acid (AA) identity. ^c^ All microarray-identified genes, with the exception of *lhpl4* and *mtco1*, were quantified by qPCR (see Materials and Methods). ^d^ Gene Ontology (GO) terms were selected from putative *Homo sapiens* orthologues (i.e., best BLASTx hit). Representative GO terms were identified (i.e., redundancies were not included), and divided into the categories: biological process (BP), molecular function (MF) and cellular component (CC). ^e^ Fold-change values between the 2 dietary treatments (high ω6/high ω3) for each of the significant microarray features. Down-regulated transcripts are shown with negative values (−(1/fold-change)) The SAM- and RP-identified *itgb5* showed fold-changes of −5.12 and −5.25, respectively.

**Table 3:** Hepatic qPCR analysis of microarray-identified transcripts, and comparison between the microarray and qPCR results.

Microarray Probe ^a^	Transcript Name	qPCR RQ Values ^b^			*p*-Value (qPCR) ^c^	Fold-Change ^d^	
		High ω3	Balanced	High ω6		Microarray	qPCR
N/A	*htra1a*	2.2 ± 0.41	1.9 ± 0.29	3.0 ± 0.65	0.25	N/A	1.34
C231R170	*htra1b*	6.0 ± 2.14	10.7 ± 3.56	22.4 ± 7.43	0.07	3.57	3.75
C103R052	*p43*	3.4 ± 0.66	2.9 ± 0.41	2.2 ± 0.47	0.24	−2.79	−1.59
C067R040	*eif2a*	5.2 ± 0.40	5.3 ± 0.74	3.5 ± 0.92	0.19	−3.13	−1.47
C253R093	*eif4b1*	8.8 ± 1.78	6.7 ± 1.43	5.5 ± 1.53	0.29	−3.23	−1.59
N/A	*eif4b2*	2.7 ± 0.30	2.7 ± 0.40	2.2 ± 0.30	0.55	N/A	−1.22
C060R108	*mtco2*	1.4 ± 0.10	1.2 ± 0.06	1.4 ± 0.14	0.43	−3.27	1.07
C159R112	*lect2a*	7.6 ± 2.58	4.0 ± 1.20	4.2 ± 0.96	0.38	−3.48	−1.79
N/A	*lect2b*	3.4 ± 0.74	3.7 ± 0.89	3.8 ± 0.61	0.96	N/A	1.12
C152R057	*rpl18*	2.0 ± 0.17	2.1 ± 0.25	2.2 ± 0.16	0.88	−4.37	1.08
C133R018	*mef2d*	1.9 ± 0.10 ^a,b^	2.0 ± 0.11 ^a^	1.5 ± 0.13 ^b^	0.03	−4.54	−1.22
C065R088	*helz2a*	2.3 ± 0.32 ^a^	1.6 ± 0.15 ^a,b^	1.5 ± 0.14 ^b^	0.04	−4.71	**−1.49**
N/A	*helz2b*	2.3 ± 0.21 ^a^	1.4 ± 0.09 ^b^	1.4 ± 0.11 ^b^	0.002	N/A	**−1.61**
C002R106	*itgb5*	2.1 ± 0.23	1.9 ± 0.08	1.6 ± 0.08	0.13	−5.25	−1.34

^a^ Refers to the identity of the probe on the 44 K array. Transcripts with no probe ID are paralogues of microarray-identified transcripts. ^b^ Mean relative quantity (RQ) ± standard error (*n* = 6–8). RQ values were normalized to *elongation factor 1 alpha-2* (*eef1α-2*) and *60S ribosomal protein 32* (*rpl32*), and calibrated to the lowest expressing individual for each gene of interest. Different letters indicate significant differences among treatments (General linear model followed by Tukey pairwise comparison). ^c^ *p*-values obtained in the qPCR study. Differences were considered statistically significant when *p* < 0.05. ^d^ Microarray and qPCR comparison of fold-changes (i.e., high ω6/high ω3). Down-regulated transcripts are negative values (−(1/fold-change)). qPCR fold-changes corresponding to GOI with significant differences between the high ω6 and high ω3 treatments are bolded.

## Data Availability

The data presented in this study are available within the article. The microarray data were submitted to NCBI’s Gene Expression Omnibus (GEO) repository (GSE139418, https://www.ncbi.nlm.nih.gov/geo/query/acc.cgi?acc=GSE139418. If required, any additional data are available on request from the authors.

## Supplementary Materials

The following are available online at https://www.mdpi.com/article/10.3390/biology10070578/s1, Table S1: Formulation and nutrient composition (%) of experimental diets fed to Atlantic salmon. This diet information was published in Katan et al. [26]. However, it is included here as this information is pertinent to the current study as well, Table S2: Lipid and FA composition (%) of experimental diets fed to Atlantic salmon. This diet information was published in Katan et al. [26]. However, it is included here as this information is pertinent to the current study as well, Table S3: Paralogues of genes involved in the qPCR, and their identity (%) over the aligned nucleotide regions, Figure S1: Alignment of the nucleotide sequences of *htra1a* (GenBank accession number NM_001141717) and *htra1b* (GenBank accession number EG831192). Conserved regions are highlighted in yellow. Alignments were performed using AlignX (Vector NTI Advance 11). Forward primers are bolded and underlined, whereas reverse primers are bolded without an underline, Figure S2: Alignment of the nucleotide sequences of *eif4b1* (GenBank accession number BT072661) and *eif4b2* (accession number DY739566). Conserved regions are highlighted in yellow. Alignments were performed using AlignX (Vector NTI Advance 11). Forward primers are bolded and underlined, whereas reverse primers are bolded without an underline, Figure S3: Alignment of the nucleotide sequences of *lect2a* (GenBank accession number BT059281) and *lect2b* (GenBank accession number DV106130). Conserved regions are highlighted in yellow. Alignments were performed using AlignX (Vector NTI Advance 11). Forward primers are bolded and underlined, whereas reverse primers are bolded without an underline, Figure S4: Alignment of the nucleotide sequences of *helz2a* (GenBank accession number BT072427) and *helz2b* (GenBank accession number EG928625). Conserved regions are highlighted in yellow. Alignments were performed using AlignX (Vector NTI Advance 11). Forward primers are bolded and underlined, whereas reverse primers are bolded without an underline.
