# Supplementary material for: Influence of Varying Dietary ω6 to ω3 Fatty Acid Ratios on the Hepatic Transcriptome, and Association with Phenotypic Traits (Growth, Somatic Indices, and Tissue Lipid Composition), in Atlantic Salmon (Salmo salar)
Source: Biology (Basel). 2021 Jun 24;10(7):578. doi: 10.3390/biology10070578 (PMC8301090; doi:10.3390/biology10070578)
Supplement: Supplementary file 1 [file biology-10-00578-s001.zip › biology-1209479-SI.pdf]

Supplementary Material

Table S1. Formulation and nutrient composition (%) of experimental diets fed to Atlantic salmon. This diet information was published in Katan et al. 2019. However, it is included here as this information is pertinent to the current study as well.

Ingredient (%)[a]	High ω3	Balanced	High ω6
Marine protein[b]	26.7	26.7	26.7
Plant protein[c]	44.6	44.6	44.6
Additives[d]	2.6	2.6	2.6
Fish oil	4.8	4.8	4.8
Linseed oil	16.7	8.3	0
Soy oil	0	9.8	19.6
Palm oil	4.6	3.2	1.7
Total (%)	100	100	100
Digestible energy (MJ Kg^{-1})	20.4	20.4	20.4
Digestible protein (g Kg^{-1})	421	421	421
Dry matter (%)[e]	94.8 ± 0.1	94.8 ± 0.1	94.8 ± 0.1
Ash (%)[e]	6.2 ± 0.04	6.0 ± 0.3	6.2 ± 0.2

[a] All ingredients were sourced from Cargill Innovation stocks.

[b] Marine protein is comprised of fish meal.

[c] Plant protein concentrate is a proprietary blend of soy, corn and wheat.

[d] Additives are proprietary mineral, vitamin mix and amino acids. Mineral and vitamin composition is proprietary information to Cargill Innovation.

[e] Analysed as % of wet weight (n = 3).

Table S2. Lipid and FA composition (%) of experimental diets[a] fed to Atlantic salmon. This diet information was published in Katan et al. 2019. However, it is included here as this information is pertinent to the current study as well.

	High ω3	Balanced	High ω6
Lipid class composition (% of total lipid)			
TAG[b]	80.0	82.9	76.9
Sterol	3.1	3.4	1.9
AMPL[c]	4.5	1.6	6.4
Phospholipid	2.6	3.3	2.7
Total lipids (mg g^{-1} wet weight)	189	215	206
FA composition (% of total FAs)			
14:0	1.7	1.5	1.6
16:0	13.7	13.0	13.2
16:1ω7	1.7	1.7	1.7
18:0	2.9	3.0	3.0
18:1ω7	1.0	1.2	1.4
18:1ω9	20.1	19.6	19.4
**18:2ω6 (LNA)**	12.7	24.6	36.2
**18:3ω3 (ALA)**	29.8	18.9	6.4
20:1ω9	2.5	2.5	2.6
20:4ω6 (ARA)	0.1	0.1	0.1
20:5ω3 (EPA)	2.6	2.6	2.6
22:6ω3 (DHA)	3.2	3.2	3.2
Σ SFA[d]	19.1	18.5	19.0
Σ MUFA[e]	29.8	29.5	29.8
Σ PUFA[f]	50.9	51.9	51.0
**Σ ω3**	37.1	26.2	13.7
**Σ ω6**	13.0	24.9	36.5
ω6:ω3	0.4	0.9	2.7
DHA/EPA	1.3	1.2	1.3
EPA/ARA	20.7	22.9	20.5
EPA+DHA (% of wet weight)[g]	1.0	1.1	1.3

[a] Mean (n=3). [b] Triacylglycerol. [c] Acetone mobile polar lipid. [d] Total saturated fatty acids. [e] Total monounsaturated fatty acids. [f] Total polyunsaturated fatty acids. [g] EPA+DHA analysed as % of dietary wet weight. Fatty acids that showed the largest range among diets are bolded.

Table S3. Paralogues of genes involved in the qPCR study, and their identity (%) over the aligned nucleotide regions.

Transcript name (symbol)	GenBank Accession no.	Aligned region (bp)	Identity (%)	E-value
Serine protease HTRA1 precursor a (*htra1a*)<br>Serine protease HTRA1 precursor b (*htra1b*)	NM001141717<br>EG831192	591	91	0
Eukaryotic translation initiation factor 4B 1 (*eif4b1*)<br>Eukaryotic translation initiation factor 4B 2 (*eif4b2*)	BT072661<br>DY739566	756	82	0
Leukocyte cell-derived chemotaxin 2 precursor a (*lect2a*)<br>Leukocyte cell-derived chemotaxin 2 precursor b (*lect2b*)	BT059281<br>DV106130	531	88	0
Helicase with zinc finger domain 2 a (*helz2a,* alias *pric285a*)<br>Helicase with zinc finger domain 2 b (*helz2b,* alias *pric285b*)	BT072427<br>EG928625	828	67	1.00e-60

```
                                 1                                                50
                                 1                                                50
htra1a_NM001141717       (1)  CAACAAAGCTCATTTTAGTGAAAAGCGTCTGACATTTATTTGCAAATGTA
   htra1b_EG831192       (1)  --------------------------------------------------
                                 51                                              100
htra1a_NM001141717      (51)  TGTAACGTTTATTTATGCAGATCAAACATATTTTTGTAGCAAGTCGTAAA
   htra1b_EG831192       (1)  --------------------------------------------------
                                 101                                             150
htra1a_NM001141717     (101)  GTTTTAAGAGTCGTTTTTTTTGGCAACTTTGTACCTTTTTAGATACTTTA
   htra1b_EG831192       (1)  --------------------------------------------------
                                 151                                             200
htra1a_NM001141717     (151)  ATATTTAACAATGTTTTGGTGCGTCTTCTGCGCAACTTTTATTCTTGCTC
   htra1b_EG831192       (1)  --------------------------------------------------
                                 201                                             250
htra1a_NM001141717     (201)  CTTTAGTTTGCGAGTCAAGAGCCAAGCGATATGTCATCGGCTGTCCAGAT
   htra1b_EG831192       (1)  --------------------------------------------------
                                 251                                             300
htra1a_NM001141717     (251)  AAATGTGACAAATTTCTATGTCCCCCGATCCCTGCGGACTGTTTGGCCGG
   htra1b_EG831192       (1)  --------------------------------------------------
                                 301                                             350
htra1a_NM001141717     (301)  CGACATCCTTGACCAATGCGACTGCTGTCCGGTCTGTGCGCACGGAGAAG
   htra1b_EG831192       (1)  --------------------------------------------------
                                 351                                             400
htra1a_NM001141717     (351)  GTGAGGTGTGCGGCGGCACGGGGAGACTAGGGGACCCGGAGTGCGGAGAG
   htra1b_EG831192       (1)  --------------------------------------------------
                                 401                                             450
htra1a_NM001141717     (401)  GGCATGGACTGCTCGATATCGGACGGAATTGGGGTGTCCGCCACAGTAAG
   htra1b_EG831192       (1)  --------------------------------------------------
                                 451                                             500
htra1a_NM001141717     (451)  GCGTCGGGGCAAAAACGGTGTGTGCGTCTGCAAAGTTGCGGACCCGGTGT
   htra1b_EG831192       (1)  --------------------------------------------------
                                 501                                             550
htra1a_NM001141717     (501)  GCGGCAGTGACGGGGTGTCCTACCGAAACATCTGCGAACTGAAGAGATTG
   htra1b_EG831192       (1)  --------------------------------------------------
                                 551                                             600
htra1a_NM001141717     (551)  AGTAACCGGGCTCTGAAGCTTCAGCAGCCACCGGTCATCTTCATACAGAG
   htra1b_EG831192       (1)  --------------------------------------------------
                                 601                                             650
htra1a_NM001141717     (601)  AGGAACCTGTAGCAAAGGCCAGGAGAATCCAGACAGTCTGCGCCACAGAT
   htra1b_EG831192       (1)  --------------------------------------------------
                                 651                                             700
htra1a_NM001141717     (651)  ATAACTTCATCGCTGATGTGGTGGAGGAGATCGCTCCCGCTGTGGTTCAT
   htra1b_EG831192       (1)  --------------------------------------------------
                                 701                                             750
htra1a_NM001141717     (701)  ATTGAACTTTACCGCAAGATGGTGTTCTCTAAGCGTGAGGTGGCGGTGGC
   htra1b_EG831192       (1)  --------------------------------------------------
                                 751                                             800
htra1a_NM001141717     (751)  CAGCGGGTCTGGCTTCGTGGTGTCAGAGGACGGCTTGATTGTGACCAACG
   htra1b_EG831192       (1)  --------------------------------------------------
                                 801                                             850
htra1a_NM001141717     (801)  CCCACGTGGTGGCCAATAAGCACCGGGTGAAGGTGGAGCTGAAGAGTGGC
   htra1b_EG831192       (1)  --------------------------------------------------
                                 851                                             900
htra1a_NM001141717     (851)  GCCACCTTCGACGCCAAGATCACAGACGTGGACGAGAAGGCAGACATTGC
   htra1b_EG831192       (1)  --------------------------------------------------
                                 901                                             950
```

```
htra1a_NM001141717   (901) CCTCATCAAGATCGACACCCCGATGAAACTGCCGGTGCTGCTGCTGGGTC
   htra1b_EG831192     (1) --------------------------------------------------
                           951                                           1000
htra1a_NM001141717   (951) GTTCAGCTGACCTGAGGCCTGGTGAGTTTGTTGTGGCCATCGGCAGCCCC
   htra1b_EG831192     (1) -------------------GGGGGAGTTTGTGGTGGCCATCGGCAGCCCC
                           1001                                          1050
htra1a_NM001141717  (1001) TTCTCCCTGCAGAACACGGTCACCACAGGTATCGTCAGCACCACCCAAAG
   htra1b_EG831192    (32) TTCTCCCTGCAGAACACGGTCACCACAGGTATCGTCAGCACCACCCAGAG
                           1051                                          1100
htra1a_NM001141717  (1051) AGGAGGCAAGGAGCTTGGCCTGAGGAACTCTGATATGGAATACATCCAGA
   htra1b_EG831192    (82) AGGAGGCAAGGAGCTGGGCCTGAGGAACTCTGATATGGACTACATCCAGA
                           1101                                          1150
htra1a_NM001141717  (1101) CGGACGCTATCATCAACGAAGGAAGTGTAGATCTACCATCTGATCATCCC
   htra1b_EG831192   (132) CGGACGCTATCATCAAC--------------------------T------
                           1151                                          1200
htra1a_NM001141717  (1151) TTTAACCTGAATGCTGTTCCTTTTCAGTATGGGAACTCTGGCGGACCCCT
   htra1b_EG831192   (150) ----------------------------ATGGGAACTCTGGCGGACCCCT
                           1201                                          1250
htra1a_NM001141717  (1201) GGTCAATCTGGACGGAGAGGTGATTGGGATCAACACACTGAAGGTGACAG
   htra1b_EG831192   (172) GGTCAATCTGGATGGAGAGGTGATTGGGATCAACACACTGAAGGTGACAG
                           1251                                          1300
htra1a_NM001141717  (1251) CAGGAATCTCCTTCGCCATCCCCTCAGACAAGATCCGTCAGTTCTTGGCA
   htra1b_EG831192   (222) CAGGAATCTCCTTCGCCATCCCCTCAGACAAGATCCGTCAGTTCCTGGCA
                           1301                                          1350
htra1a_NM001141717  (1301) GAGTCCCACGGCAGACAATCTAAAGGTAGA--------------------
   htra1b_EG831192   (272) GAGTCCCATGACAGGCAATCTAAAGGTACAGATTACCAATCTAAAGGGAA
                           1351                                          1400
htra1a_NM001141717  (1331) -TTATTACCAAAGAAGAAGTATATCGGTGTGAGGATGATGACTCTCACTA
   htra1b_EG831192   (322) ATTATCAACAAGTAAGAAATATATCGGTGTGAGGATGATGACTCTCACAC
                           1401                                          1450
htra1a_NM001141717  (1380) CAACGCTTGCAAAGGAGCTGAAGGAGAGAACATCAGACTTCCCTGATGTT
   htra1b_EG831192   (372) CAATGCTTGCAAAGGAGCTGAAGGAGAGACAATCAGACTTCCCCGATGTT
                           1451                                          1500
htra1a_NM001141717  (1430) ACCTCAGGGGCATATGTCATCGAGGTCATCCCAAAAACACCAGCTGAGAC
   htra1b_EG831192   (422) ACCTCAGGGGCATATGTAATCGAGGTCATCCCAAAAACACCAGCTGAGAC
                           1501                                          1550
htra1a_NM001141717  (1480) AGGTGGCCTGCAGGAGAGTGACGTCATAATCACCATCAACAGCCAGCGAA
   htra1b_EG831192   (472) AGGTGGCCTGCAGGAGAGTGATGTCATAATCACCATCAACAGCCAGCGAA
                           1551                                          1600
htra1a_NM001141717  (1530) TCACCTCGGCCAGTGATGTCAGCAGCTCTATCAAGAGGGACGACACGCTG
   htra1b_EG831192   (522) TCACCTCAGCCAGTGACGTCAGCAGCTCCATCAAGAGGGAAGACACGCTG
                           1601                                          1650
htra1a_NM001141717  (1580) CGAATGGTGGTCCGGCGGGGGAACGAGGACATCATGCTCACCGTCGTCCC
   htra1b_EG831192   (572) CGCATGGTGGTCCGGCGGGGGAACGAGGACGTCATGCTCACCGTCGTCCC
                           1651                                          1700
htra1a_NM001141717  (1630) CGAGGACATTGACCCTTGACCTCTCAGCAACCACGAGCTGGTTCTCAGTG
   htra1b_EG831192   (622) AGAGGAGATTGACCCTTGACCTCTCAGCAACCACGAGCTGGTTCTCAGTG
                           1701                                          1750
htra1a_NM001141717  (1680) TTTAAAACCACGGAC-TTAAACCGGTTGTGTGTCTGGATCCACACCCTAC
   htra1b_EG831192   (672) TTGAAAATCACGGACCTTAAACCGG--GTGTGTCTGGAGCCACACCCTAC
                           1751                                          1800
htra1a_NM001141717  (1729) CATAGAACCTGTAACTACGGGTG----CTCTTCGCTCTCAGTGAA--ACT
   htra1b_EG831192   (720) CTTAGAACCTGTAACTACTAGTGTCTCCCCCCACTCCCCTCAAACTACA
                           1801                                          1850
htra1a_NM001141717  (1773) GATTATTGTTTTATACTCTACTAACGTCCTTGCACAGAAAACCCACCTCA
   htra1b_EG831192   (770) GAGGTCTGTGAGGCCCACAACTAACCCTCTGGGAGCTCAAATGTGT----
                           1851                                          1900
```

```
htra1a_NM001141717  (1823) ATCAGATAGAAAAGTGGGTGTCACAGATTACCGTAGTTTTGTAGTTGTTT
   htra1b_EG831192   (816) --------------------------------------------------
                           1901                                            1950
htra1a_NM001141717  (1873) TTGTTGCTGTTGATATTATGTTTTTTTTTTATTGTATTTTAATTGCATTT
   htra1b_EG831192   (816) --------------------------------------------------
                           1951                                            2000
htra1a_NM001141717  (1923) CTCTTGATACGTTTTGACGACAATTTCACAGAGGGAATAAAAAGATTTTG
   htra1b_EG831192   (816) --------------------------------------------------
                           2001                     2027
htra1a_NM001141717  (1973) AAAAAAAAAAAAAAAAAAAAAAAAAGA
   htra1b_EG831192   (816) ---------------------------
```

Figure S1. Alignment of the nucleotide sequences of *htra1a* (GenBank accession number NM_001141717) and *htra1b* (GenBank accession number EG831192). Conserved regions are highlighted in yellow. Alignments were performed using AlignX (Vector NTI Advance 11). Forward primers are bolded and underlined, whereas reverse primers are bolded without an underline.

```
                        1                                                  50
```

```
eif4b1_BT072661      (1) ATAAGAAGGGGAAGACCCTGACCCTCACTGACTTCCTGGCAGAGGACAAT
eif4b2_DY739566      (1) --------------------------------------------------
                         51                                             100
eif4b1_BT072661     (51) GGGAGTGGAGGCAATGCTCCACCACCGCAACCCAGCTACGCCAAGTCCAC
eif4b2_DY739566      (1) --------------------------------------------------
                         101                                            150
eif4b1_BT072661    (101) CAGCTGGGCCGACGAGACGGATGACCTGGAGGGAGATGTGTCCACCTCCT
eif4b2_DY739566      (1) --------------------------------------------------
                         151                                            200
eif4b1_BT072661    (151) GGCACTCAGGGGAGGACAGTTACCGGGCCCCGGCCATAGATCGCAACATC
eif4b2_DY739566      (1) --------------------------------------------------
                         201                                            250
eif4b1_BT072661    (201) CTGCCCACGGCGCCACGGTCGGCCCGCGAGCCCAATGTGGACCGGTCACG
eif4b2_DY739566      (1) --------------------------------------------------
                         251                                            300
eif4b1_BT072661    (251) CCTCCCCCGCAGCCCCCCCTACACCGCCTTCCTGGGCAACCTGCCCTACG
eif4b2_DY739566      (1) --------------------------------------------------
                         301                                            350
eif4b1_BT072661    (301) ACGTCTCCGAGGAATCTATCATGGACTTCTTCCGGGGCCTAGCGATCAGT
eif4b2_DY739566      (1) --------------------------------------------------
                         351                                            400
eif4b1_BT072661    (351) GCTGTGCGCTTGCCACGGGAGCCCAGTAACCCAGAGAGGCTGAAGGGCTT
eif4b2_DY739566      (1) --------------------------------------------------
                         401                                            450
eif4b1_BT072661    (401) TGGCTACGCAGAGTTTGATGATGTGGACTCCCTCCTGAGGGCGCTGACTC
eif4b2_DY739566      (1) --------------------------------------------------
                         451                                            500
eif4b1_BT072661    (451) TCAATGAGGAGAACCTGGGAAACCGCAGGATCCGGGTGGATATTGCAGAT
eif4b2_DY739566      (1) --------------------------------------------------
                         501                                            550
eif4b1_BT072661    (501) CAGTCCAACGACAAGGAGGGGAGAGATAATGGCCAGATGGGTGGACGGGA
eif4b2_DY739566      (1) --------------------------------------------------
                         551                                            600
eif4b1_BT072661    (551) CAGGATGGGCCGTATGGGAGACATGGGGGGCCCCGACAAGACAGACAGTG
eif4b2_DY739566      (1) --------------------------------------------------
                         601                                            650
eif4b1_BT072661    (601) ACGACTGGAGGGCCCGGCCCACTGCTGACGCTGATGACGGACCCCCAAAG
eif4b2_DY739566      (1) --------------------------------------------------
                         651                                            700
eif4b1_BT072661    (651) AGAGAGGAATCCACTTTCGGGTCACGCGACCGCTATGGAGACCGTGACGG
eif4b2_DY739566      (1) --------------------------------------------------
                         701                                            750
eif4b1_BT072661    (701) GCTGAGACGGGACAACGACCGCGGATTTGGCGGCGACCGGGAC-CGCGGA
eif4b2_DY739566      (1) ------------------------------CCGCCTTCGGGTCACGCGAC
                         751                                            800
eif4b1_BT072661    (750) TTTGGCGGCGACCGGGACCGCGGATTTGGCGGCGACCGGGACCGCGGATT
eif4b2_DY739566     (21) CGCTACGGAGACCGTGA---CGGGCTGCGAAGGGACAACGACCGCGGCTT
                         801                                            850
eif4b1_BT072661    (800) TGGCGGCGACCGGGACGGCGGGAGAGACCGCGGGTTCGGTGGCAGAGACC
eif4b2_DY739566     (68) CGGCGGCGACCGGGAC------------CGCGGCTTCGGCGGCAGAGACC
                         851                                            900
eif4b1_BT072661    (850) GCTATGATGACCGGGGAGGTGAGAGCGGAGCCTTTGGCTCCCGCAGGGAC
eif4b2_DY739566    (106) GCTATGACGACCGGGGAGGTGAGAACGGAGCCTTTGGCTCCCGCAGGGAT
                         901                                            950
eif4b1_BT072661    (900) CGGGATGATGGCGGGCGACGTGCCTTTGGCAGTGGCTACCGCCGTGATGA
eif4b2_DY739566    (156) AGGGATGACGGCGGGCGACGTGCCTTCGGCAGTGGGTACCGCCGTGACGA
                         951                                           1000
```

```
eif4b1_BT072661    (950)  TGAC------------GGGGGTGGTGGTGGCCGCTACGGGGAACGGGATC
eif4b2_DY739566    (206)  TGACTATCAGAGTGGCGGAGGTGGCGGTAGCCGCTATGGGGCCCGGGATC
                          1001                                          1050
eif4b1_BT072661    (988)  GCTACGGCGCGGACAGAGAGGACCGATATGAGAGGCGCGAGGA-GAGAG-
eif4b2_DY739566    (256)  GCTATGGCGGAGACCGGGAGGGCCGGTATGAGAGGCAGGACGAAGGGCGT
                          1051                                          1100
eif4b1_BT072661   (1036)  GAGGTGAG-GGTGGTCCCACGCAGAGACCCAAGCTGGTCCTGAAGCCACG
eif4b2_DY739566    (306)  GAGGAGAGAGGTGGTCCGATCGAAAGGCCCAAGTTGGTCCTGAAGCCGCG
                          1101                                          1150
eif4b1_BT072661   (1085)  CAGCACGCCCAAGGAGGAGGAGCAGGCCCGCAGCGGTGGTGCGGGAGCTG
eif4b2_DY739566    (356)  CAGCATGGCCAAGGAGGAGCAGCAGGCCGGCAGCGGTGGGGC------TA
                          1151                                          1200
eif4b1_BT072661   (1135)  CCCCAGCTGCTCCAGCGACTGCCCCCAGCTCCGGCCGTGCCTCCATCTTC
eif4b2_DY739566    (400)  C---------TCCGGCAGCCGCCCGCAGCTCTGGTCGAGCCTCCATCTTC
                          1201                                          1250
eif4b1_BT072661   (1185)  GGAGCAGCCAAGCCCGTGGACACTGCAGCCAAGGAGCGGGAGGTGGAGGA
eif4b2_DY739566    (441)  GGAGCGGCCAAGCCCGTGGACACAGCGGCTAAGGAGCGGGAGGTGGAGGA
                          1251                                          1300
eif4b1_BT072661   (1235)  GAAACTCCAGAGGCAGCTGGAAGAGGACAAGTCCAGGGGCTTTGATAGGA
eif4b2_DY739566    (491)  GAAGCTCCAGAGGCAGCTGGAAGAGGACAAGTCCAAGGGCTTCGATAGGA
                          1301                                          1350
eif4b1_BT072661   (1285)  AACCCCGCGATAGAGACAGGGACCCAAGTTGGAGGAGTGAGGAGCCACCT
eif4b2_DY739566    (541)  AACCTCGCGACAGAGACAGGGACCCAAGCTGGAGGAATGAGGAGCCACCT
                          1351                                          1400
eif4b1_BT072661   (1335)  TCTGAGCGACCTGCTACACGCTCCCGCACAGGAAGTGAGTCATCACAGAC
eif4b2_DY739566    (591)  TCTGAGC------------GGTCCCGCACAGGAAGTGAGTCATCACATCC
                          1401                                          1450
eif4b1_BT072661   (1385)  GGGAAGTACATCTGGAGGAAGAGTCTCGCGGCGCAGAGAGAGCGAGCGAT
eif4b2_DY739566    (629)  AGGAAGTACCTCTGGAGGGAGAGCCTCACGGCGCAGAGAGAGCGAGCGCT
                          1451                                          1500
eif4b1_BT072661   (1435)  CGGTTGAGAATGAAGTATTCAGTGGTAAGGAGGATGACCCTCCCTCTCCC
eif4b2_DY739566    (679)  CAGTGGAGAACGAAGTATTCAGCGGTAAGGAGGACGACCCTCCCTCTCCC
                          1501                                          1550
eif4b1_BT072661   (1485)  GGAGCTCGCCCTACTTCCGCCAACTCTTCCACATCTTCCTCCAAGGAGCC
eif4b2_DY739566    (729)  GGGTCTCGCCCCTCTTCCGCCAACTCTTCCACCTTCTCCTCCAAG-----
                          1551                                          1600
eif4b1_BT072661   (1535)  CCTCAAGGTGATGCCCGCACCACCACCCAAGGAGAATGCCTGGGCCAAGC
eif4b2_DY739566    (774)  --------------------------------------------------
                          1601                                          1650
eif4b1_BT072661   (1585)  GCCCTGCGGTGAGCGCAGGGTCCACCCCTGTCTCCCCCAGTGACGCGGCA
eif4b2_DY739566    (774)  --------------------------------------------------
                          1651                                          1700
eif4b1_BT072661   (1635)  TGTCCCAAACTGAGCTCAAGTTCTGCAGATGAAAGAGGATCTGGAAGGGA
eif4b2_DY739566    (774)  --------------------------------------------------
                          1701                                          1750
eif4b1_BT072661   (1685)  AATTCAGCTCAGCCAGTAAATACGCCGCTTTGCTAATGGACAGCGAGCAA
eif4b2_DY739566    (774)  --------------------------------------------------
                          1751                                          1800
eif4b1_BT072661   (1735)  GGAGACGACGCAGAGGACAGCGTAGAATAAAGCGAGAGCGGGGGAGTGAA
eif4b2_DY739566    (774)  --------------------------------------------------
                          1801                                          1850
eif4b1_BT072661   (1785)  AAGAAGATCCAGCCAATGAAAGAATCTCACATTAAATATGAAAGTCTCCT
eif4b2_DY739566    (774)  --------------------------------------------------
                          1851                                          1900
eif4b1_BT072661   (1835)  CACCTGGTGTAGAGGCACCTGGGAGGTCATCACTTCTACCTACTTTCCTT
eif4b2_DY739566    (774)  --------------------------------------------------
                          1901                                          1950
```

```
eif4b1_BT072661  (1885) ACTCGTAACCCTCCTTCCCCTTTTCAGGACTTTTCAGTTTCTAAAAAGAA
eif4b2_DY739566   (774) --------------------------------------------------
                        1951                                          2000
eif4b1_BT072661  (1935) TAGTCATATACTGACTTGTTTTAAAATGGAAAAGGAAAGGAATAGTCGAC
eif4b2_DY739566   (774) --------------------------------------------------
                        2001                                          2050
eif4b1_BT072661  (1985) ACAAGAAAATATGGAAAATTGAATTAATTGTAATAAATAAAAAAAGTGGG
eif4b2_DY739566   (774) --------------------------------------------------
                        2051                                          2100
eif4b1_BT072661  (2035) GTAAAAACAAATTGTTTACATTTGTGATGGTATGAGTTGGGCTCCATCCT
eif4b2_DY739566   (774) --------------------------------------------------
                        2101                                          2150
eif4b1_BT072661  (2085) TCTCCCCAGTGGAAAAGGATGAAGAGTTGGTTACTGGATGCTTGCTTGTC
eif4b2_DY739566   (774) --------------------------------------------------
                        2151                                          2200
eif4b1_BT072661  (2135) AGCCTGACCTATTAAATCTCATGTGCAGTTAAAAAAAAAAAAAAAAAAAA
eif4b2_DY739566   (774) --------------------------------------------------
                        2201
eif4b1_BT072661  (2185) AAAAAAAAGA
eif4b2_DY739566   (774) ----------
```

Figure S2. Alignment of the nucleotide sequences of *eif4b1* (GenBank accession number BT072661) and *eif4b2* (accession number DY739566). Conserved regions are highlighted in yellow. Alignments were performed using AlignX (Vector NTI Advance 11). Forward primers are bolded and underlined, whereas reverse primers are bolded without an underline.

```
lect2a_BT059281      (1) -CAAGACTTTCTTGTAGATTTTCTCCTTGA-GTGAAAGCATCATCTAAGG
lect2b_DV106130      (1) GCAAGACCTTCTTGCAGAGTTTTTCCTTGAAGCGAAAGCATCCTCTCAGG
                         51                                             100
lect2a_BT059281     (49) CTTTACCATGAGGACTGCTGTTCTTTTGTTTACTGTGGTGCTCATAGCTG
lect2b_DV106130     (51) CTTCAACATGAAGACAGCTATTCTTCTGCTTACTGTAGTCCTTATGGCTG
                         101                                            150
lect2a_BT059281     (99) TGTTGTCAGAGTGCGAGATGGTCAAGTTTGGTCAGCTGTGCAGCGGCAAC
lect2b_DV106130    (101) TGTTGCCAGAGTGTGAGATGGCCAAGTTTGGTCAGCTGTGTAGCGGAAAT
                         151                                            200
lect2a_BT059281    (149) TCCAGTAACAGGAGGAGGACAGGGGACAGATGGGGACAAGGACACTACGG
lect2b_DV106130    (151) TCCAGTAACAGGAGGAGGACAGGGGACAACTGGGGACAAGGACAGTACGG
                         201                                            250
lect2a_BT059281    (199) CGCACGCAGAGGAAACCGTGAGCATCAGGGCCTGGACATTGTGTGTAATG
lect2b_DV106130    (201) AGCAAGCAGAACAGACCATGTGCATAAGGGCATTGACATCGTGTGTAACG
                         251                                            300
lect2a_BT059281    (249) ATGGGGCCACAGTGTACGCTCCATTTGATGTGAAACTCAATGGGAAAGTG
lect2b_DV106130    (251) ACGGGGCCACAGTGTACGCTCCATTTGACGTGAAACTCAACGGCAAAGTG
                         301                                            350
lect2a_BT059281    (299) ATCGTGTACACAGACCCGAAGAAGGCAGCCATCAATGATGGGATCAACCT
lect2b_DV106130    (301) ACAGTGTACACAAACCCAAAGAAAGCAGCCATCAACGATGGGATCAACCT
                         351                                            400
lect2a_BT059281    (349) CAGTGGAGAGGGTCTGTGCTTTAAGCTGTTCTACGTAAAGCCTGACAAGT
lect2b_DV106130    (351) CAGTGGGGAAGGTCTGTGCTTTAAGCTGTTCTACGTGAAGCCTGACAGTT
                         401                                            450
lect2a_BT059281    (399) ACTCTGGGGTGGTGAAGAAGGGCCAGAGGATTGGGACCCTGCTGACCATG
lect2b_DV106130    (401) ACTCTGGGGTGGTGAAGAAGGGCCAGAGGATTGGGACCCTGCTCCCCATG
                         451                                            500
lect2a_BT059281    (449) CAAAGTGTCTACCCAGGGATCACTTCTCACGTCCACGTCCAGATGTGTGA
lect2b_DV106130    (451) CAGAGTGTCTACCCAGGGATCACTTCTCACGTCCACGTCCAGATGTGTGA
                         501                                            550
lect2a_BT059281    (499) CAAGTCTGACCCCACCAAGTTTTTCTAATGGAGTCCCCTTTGGCCTCTCC
lect2b_DV106130    (501) CAAGTCCGACCCCACCAAGTACTTCTGATTGA-------TTGG---TTAA
                         551                                            600
lect2a_BT059281    (549) ATCAATCAATCAATCAATCAATCAATCAATCAATCAATCAATCAATCAAT
lect2b_DV106130    (541) ATAAATCATCTAAATTATCAC-CAATG-----------------------
                         601                                            650
lect2a_BT059281    (599) CAATCAATCAATCAATCAATCAATCAATCAATCAATCAATCTACAAAATG
lect2b_DV106130    (567) --------------------------------------------------
                         651                                            700
lect2a_BT059281    (649) ATTGTAATCATTGGCCAATAGATGGGCTTACTGTGTTTAAAAATAATAAT
lect2b_DV106130    (567) --------------------------------------------------
                         701                                            750
lect2a_BT059281    (699) TTGCTTATTATAATAAACATTTTTTATTACAGTATAAAATACATAAAAGT
lect2b_DV106130    (567) --------------------------------------------------
                         751                                            800
lect2a_BT059281    (749) TGCACACTTCTGGAATAAAGTTTTAACCCTTTAAGCATCAACAGGGGTAA
lect2b_DV106130    (567) --------------------------------------------------
                         801                                            850
lect2a_BT059281    (799) TTCATAATTTCAAAAACCTTTTTTTATCCTTTTATGCAACTAGTTACAG
lect2b_DV106130    (567) --------------------------------------------------
                         851                                            900
lect2a_BT059281    (849) ACATGAAAATAATTTACAATGATTTCTGTGTTAATATATAATTTCTGTGG
lect2b_DV106130    (567) --------------------------------------------------
                         901                                            950
lect2a_BT059281    (899) TATATATATATATATATATATATATATATATATATATATATATATATATA
lect2b_DV106130    (567) --------------------------------------------------
                         951                                           1000
```

```
lect2a_BT059281   (949) TAAAATCTCTTTTTTGTGTACTAGTTTTACGTGGCTAATCTGACAATGTG
lect2b_DV106130   (567) --------------------------------------------------
                        1001                                          1050
lect2a_BT059281   (999) GACTCAATAGTAAACTTTGAAAAAAACATAGATTTAAAACAAATACACGT
lect2b_DV106130   (567) --------------------------------------------------
                        1051                                          1100
lect2a_BT059281  (1049) GTCAAGGCTGTGGGTAACTGGTGAAAGGAGTCAGGCGCAGGAGAGCTGAG
lect2b_DV106130   (567) --------------------------------------------------
                        1101                                          1150
lect2a_BT059281  (1099) ATGCGTGGACAAGGTATTTAATACAAGAAAACATCAGTATGAACACAATA
lect2b_DV106130   (567) --------------------------------------------------
                        1151                                          1200
lect2a_BT059281  (1149) CTATGGTGCTGGAAAAAAAACGGTACCACGAAATTAACAGGCGTAATAAA
lect2b_DV106130   (567) --------------------------------------------------
                        1201                                          1250
lect2a_BT059281  (1199) AAAACCCGGTAACAATATACCAGCCGTCAGATACAGCCTTACAATAAAAC
lect2b_DV106130   (567) --------------------------------------------------
                        1251                                          1300
lect2a_BT059281  (1249) AAAGACGCACACAAACATCGGGGAAACCAGAGGGTTAAATAATGAACATG
lect2b_DV106130   (567) --------------------------------------------------
                        1301                                          1350
lect2a_BT059281  (1299) TAATGGGGGAATTGAAACCAGGTGTGTAAAAAAACAACAACAAAACAAAT
lect2b_DV106130   (567) --------------------------------------------------
                        1351                                          1400
lect2a_BT059281  (1349) GGAAAATGAAAAGTGGATCGGTGATGGCTACCGCCGAATGCCGCTCGAAC
lect2b_DV106130   (567) --------------------------------------------------
                        1401                                          1450
lect2a_BT059281  (1399) AAGGAGCGGGACCGACTCCGGCGGAAGTCGTGACAACATGTGTGTCATTG
lect2b_DV106130   (567) --------------------------------------------------
                        1451                                          1500
lect2a_BT059281  (1449) AACAACCCCATACCATGACTAGATGGTGGAAAAAAACACCCATCTCTTTA
lect2b_DV106130   (567) --------------------------------------------------
                        1501                                          1550
lect2a_BT059281  (1499) ATAAGTTCTTTAGACAATTAAAGCTAATTTACCAACATTTCTGAAAATTT
lect2b_DV106130   (567) --------------------------------------------------
                        1551                                          1600
lect2a_BT059281  (1549) GGAATGAAACTTCTATTGATAAAAATAACTGTTTAAAACATGAAAAACAT
lect2b_DV106130   (567) --------------------------------------------------
                        1601                                          1650
lect2a_BT059281  (1599) ACTAAAATGTATATTTTTAAACATGAAATTTGACTATAAATGTGTGAAAA
lect2b_DV106130   (567) --------------------------------------------------
                        1651                                          1700
lect2a_BT059281  (1649) TTTAAAATATATGCATATTTTGGGGAATTTAATAAAAGAAAAAGTGTTAC
lect2b_DV106130   (567) --------------------------------------------------
                        1701                                          1750
lect2a_BT059281  (1699) TGTTTGACAGAAAATGCCATTTTGCCATTATTTCCAATTTGGGGTTAAAA
lect2b_DV106130   (567) --------------------------------------------------
                        1751                                          1800
lect2a_BT059281  (1749) TGACCCATGTTGATGCATTTAGGGGTCAAAATATGTTGGTCCTTTTGGGG
lect2b_DV106130   (567) --------------------------------------------------
                        1801                                          1850
lect2a_BT059281  (1799) TATTAACAACAAAATAAATACATCATAAATCATTCCTAATAAATAATGTG
lect2b_DV106130   (567) --------------------------------------------------
                        1851                                          1900
lect2a_BT059281  (1849) TATTTTTGTAAAAAAAAAAAAAAAAAAAAAAAAAAAAAAAAAAAAAAAAA
lect2b_DV106130   (567) --------------------------------------------------
                        1901        1914
```

```
lect2a_BT059281  (1899) AAAAAAAAAAAAGA
lect2b_DV106130   (567) --------------
```

Figure S3. Alignment of the nucleotide sequences of *lect2a* (GenBank accession number BT059281) and *lect2b* (GenBank accession number DV106130). Conserved regions are highlighted in yellow. Alignments were performed using AlignX (Vector NTI Advance 11). Forward primers are bolded and underlined, whereas reverse primers are bolded without an underline.

```
                              654                                              703
helz2a_BT072427    (651)  CCTGAAGCCTTCATCCCCCTTGTGACCCATAAACCTGAACAGATTGTTTT
helz2b_EG928625    (199)  CCTCAGGCCCTCATCCCACTGGTCAGCAACAAACCAGAAAAGATTGTGTT
                              704                                              753
helz2a_BT072427    (701)  ACTCGGCGATCACAAGCAATTGCAGCCCATCACGCACAGTGATCTT-TCA
Helz2b_EG928625    (249)  GCTCGGTGACCACAAACAACTACGTCCCATCATCAAGAATGAACTTGTGA
                              754                                              803
helz2a_BT072427    (750)  GCAAGGTTGGGTATGAGGAAATCTCTGTTTGAGCGTTACATGGAAA----
Helz2b_EG928625    (299)  GGAAG-TTGGGGATGTCAAAGTCTCTGTTTGAGCGCTACTTTGAACGCCG
                              804                                              853
helz2a_BT072427    (796)  -----AGGCGTTGATGCTGGATACGCAGTACAGAATGCATGAGCGCATTT
helz2b_EG928625    (348)  GTCCCAGACAGTGATGCTGGATACCCAGTACAGAATGCATGAAGATATAT
                              854                                              903
helz2a_BT072427    (841)  GTGAGTTTCCTTCAAAGGAGTTCTACAACGGTATTCTCAAGACTGGAGCA
helz2b_EG928625    (398)  GCAAGTTCCCATCAGAGGAGTATTATGAAGGAAAACTGAAGACTGACGTG
                              904                                              953
helz2a_BT072427    (891)  ACTCCGAAAGACAGTGTCCTGCTCGCTCAGTCTCATCAT-CTGACGCCCA
helz2b_EG928625    (448)  GATCGCCCAAATGGTGTCCTTCAGGCTGA----CAGCAGGCAGACGCACA
                              954                                             1003
helz2a_BT072427    (940)  TCCTTTTTGGACACGTTTCCGGAAAAGAGATCAGTCTTGTGGTCTCCACC
helz2b_EG928625    (494)  TTGTTTTCGGCAACGTCAGTGGAGAAGAGGTCAGCCTGGTTGTGAGCACA
                              1004                                            1053
helz2a_BT072427    (990)  GAACGGGGGAATGAGAACTCAAAGGCCAACTCGGCAGAGGCTGAGGAATC
helz2b_EG928625    (544)  GAAAAAGGAAATGAAAACTCCAAAGCGAATATGAAGGAGAGAGACGTAGT
                              1054                                            1103
helz2a_BT072427   (1040)  GGTTCGCATCGCCTCTCTGTTGATCAAACATGCTGGGGTGGCGGCTAGCG
helz2b_EG928625    (594)  GGTTCGGATCGCCAACCAGCTGGTGACAGAATCCAAAATAAAA-CAACAG
                              1104                                            1153
helz2a_BT072427   (1090)  AC-ATAGCTATTCTGACGCCATACAATGCCCAGGTAGCCAAGGTAAACGA
helz2b_EG928625    (643)  AGTATGGCGATCCTATCTCCCTACAATGCCCAGGTGGCCGAAATTAAGAA
                              1154                                            1203
helz2a_BT072427   (1139)  AACCCTGTTAATGAAGCATATCCAGAACGTCAACGTGAATACCATCACAA
helz2b_EG928625    (693)  AGAACTGAGAAAATTAAAACTGGACGAAATCACGGTCACCACCATCACAA
                              1204                                            1253
helz2a_BT072427   (1189)  AGAGTCAAGGAAGTGAATGGCGTTATGTCATCTTGTCTACTGTGCGCTCT
helz2b_EG928625    (743)  AAAGTCAAGGAAGTGAATGGCGCTATGTCATCCTGTCCACTGTGCGCTCC
                              1254                                            1303
helz2a_BT072427   (1239)  TGTCCCAAGTCGGAAATTGACACAGAGCCTACCAAAGCATGGCTCACCAA
helz2b_EG928625    (793)  TTACCCAGCAAGGAGATCGAGACAGAGCCGGACAGGGCCTGGTTGTCCAA
                              1304                  1327
helz2a_BT072427   (1289)  GAAGCTTGGTTTTGTCATGGACCC
helz2b_EG928625    (843)  ACATGTGGGCTTTGTTCGTGACCC
```

Figure S4. Alignment of the nucleotide sequences of *helz2a* (GenBank accession number BT072427) and *helz2b* (GenBank accession number EG928625). Conserved regions are highlighted in yellow. Alignments were performed using AlignX (Vector NTI Advance 11). Forward primers are bolded and underlined, whereas reverse primers are bolded without an underline.
